# Plasma neurofilament light as a potential biomarker for cognitive decline in a longitudinal study of middle-aged urban adults

**DOI:** 10.1038/s41398-021-01563-9

**Published:** 2021-08-21

**Authors:** May A. Beydoun, Nicole Noren Hooten, Hind A. Beydoun, Ana I. Maldonado, Jordan Weiss, Michele K. Evans, Alan B. Zonderman

**Affiliations:** 1 Laboratory of Epidemiology and Population Sciences, NIA/NIH/IRP, Baltimore, MD USA; 2 Department of Research Programs, Fort Belvoir Community Hospital, Fort Belvoir, VA USA; 3 Department of Psychology, University of Maryland, Baltimore County, Catonsville, MD USA; 4 Department of Demography, University of California, Berkeley, Berkeley, CA USA

**Keywords:** Human behaviour, Predictive markers

## Abstract

Plasma neurofilament light (NfL) is a marker for neurodegenerative diseases. Few studies have examined the association of NfL with middle-aged changes in cognitive performance, and no studies have examined differential NfL effects by race. Using data from the Healthy Aging in Neighborhoods of Diversity across the Life Span (HANDLS) study (*n* = 625, Agev1: 30–66 y, 41.6% male, 56.3% African American, 27.8% below poverty), we investigated the associations of initial NfL levels and annualized change with cognitive performance over time in global mental status, verbal and visual memory, fluency, attention, and executive function. We used ordinary least squares and mixed-effects regressions stratified by race, while exploring differential associations by age group, sex, and poverty status. Over a mean follow-up of 4.3 years, we found initial NfL level was associated with a faster decline on normalized mental status scores in Whites only and in those >50 years old. Annualized increase in NfL was associated with a greater decline in verbal fluency in men. In other exploratory analyses, annualized increase in NfL was associated with a slower decline in verbal memory among individuals living above poverty; in the older group (>50 years), first-visit NfL was linked with better performance at baseline in global mental status and verbal memory. In summary, first-visit NfL was primarily associated with the global mental status decline among Whites, while exhibiting inconsistent relationships in some exploratory analyses. Plasma NfL levels can be detected and quantified in non-demented middle-aged adults and changes can be analyzed over time. More longitudinal studies are needed to address the clinical utility of this biomarker for early cognitive defects.

## Introduction

When axons become damaged, cytoskeletal proteins known as neurofilaments are released into the extracellular space, followed by the cerebrospinal fluid (CSF), with marked transmigration into the blood at a lower concentration [1]. Notably, among biomarkers for neurodegenerative disease, there is a need for minimally invasive, readily available, cost-effective biomarkers as current methods rely on measures derived from CSF and neuroimaging. Recently, sensitive methods were developed to measure blood-levels of neurofilament light (NfL) [2]. This methodological development for assaying plasma NfL has stimulated potential opportunities for large-scale applications in clinical practice and in randomized clinical trials as a method for identifying patients at risk for dementias, including Alzheimer’s disease (AD) [3]. Thus far, NfL reflects sub-cortical large-caliber axonal degeneration [4, 5]. Plasma NfL levels correlate strongly with CSF NfL levels [3, 6], adding to its clinical utility in differential diagnoses for dementias. While most studies have focused on plasma NfL’s positive association with AD, including at earlier stages [7–10], as well as other neurodegenerative diseases [11–14]. Thus, plasma NfL is a marker of non-specific neurodegeneration.

To date, only few studies have been conducted thus far reporting its predictive value for future cognitive decline and brain aging [15–22], and none have tested associations differentially across racial groups. Furthermore, few studies have examined how longitudinal changes in plasma NfL are related to change in cognition over time (e.g., [21]). Thus, our study (i) examined baseline NfL in relation to baseline and change in cognitive performance over time; (ii) examined change in NfL in relation to cognitive performance over time; (iii) examined baseline and change in NfL in relation to follow-up cognitive performance; and, (iv) tested racial differentials in those main associations; as well as exploring those associations across sex, age group, and poverty status.

## Materials and methods

### Database

We selected a sample from the Healthy Aging in Neighborhoods of Diversity across the Life Span (HANDLS) study. Since 2004, HANDLS is an on-going prospective cohort study of socioeconomically diverse White and African American adult women and men residing in Baltimore, MD. Initial data (visit 1) were collected between 2004 and 2009, in two phases. Phase I consisted of a home visit, with information collected for screening, recruitment, and a household in-person interview that included the first 24 h dietary recall of that visit. Phase II (v_1_) was performed as an in-person complete physical health examination including a cognitive test battery inside Medical Research Vehicles (MRV) and included a second 24 h dietary recall. Participants were invited for follow-up in-person visits (v_2_) between 2009 and 2013, which applied a similar protocol as v1 (phase II). Fasting blood samples were obtained from consenting participants in both in-person examinations. All participants provided written informed consent. The Institutional Review Board of the National Institutes of Health, National Institute of Environmental Health Sciences approved the HANDLS study protocol.

### Study sample

**Fig. 1:**
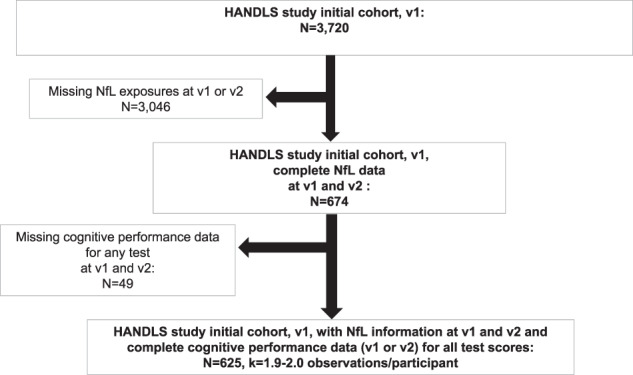
Participant flowchart. Abbreviations: *HANDLS* Healthy Aging in Neighborhoods of Diversity across the Life Span; *k* = # of observations/participant; *NfL* neurofilament light; *v*_*1*_ visit 1; *v*_*2*_ visit 2.

In our present study, up to two repeats on cognitive tests were available from v_1_ or v_2_. Exposure data on plasma NfL concentrations were available at both visits for a sub-sample of Whites and African Americans after excluding participants who did not survive within a year of follow-up or who did not have NfL data at v_2_. As shown in the study design flowchart (Fig. 1), among 3,720 initially recruited HANDLS participants, *N* = 674 had complete v_1_ and v_2_ data on plasma NfL. Of those participants, *N* = 625 had data on v_1_ or v_2_ for all 11 cognitive test scores, with an average number of observations/participant *k* = 1.9−2.0, indicating 0–5% missingness on cognitive test performance outcomes. A sub-set of those participants had complete and credible v_2_ cognitive performance data, with somewhat variable sample sizes. This sub-set was also analyzed, thus excluding those with unavailable or non-credible v_2_ cognitive performance on each test. Mean ± SD follow-up time for the final analytic sample (*n* = 625 participants) was 4.30 ± 0.95 y. Method S1 shows a detailed description for sample selection with respect to the NfL exposure. Compared to the initial sample with incomplete data for our analysis, the final sample had a lower proportion of individuals living below poverty (27.8% vs. 43.9%, *p* < 0.001, χ^2^ test), and a reduced proportion of men (41.6% vs. 45.9%, *p* = 0.048, χ^2^ test). A similar pattern was observed when the sample with v_1_ NfL (*N* = 674) was compared with the sample without this data, notwithstanding other exclusions.

### Cognitive assessment

HANDLS clinical staff examined cognitive performance with a battery of tests which included the Mini-Mental State Examination (MMSE), the California Verbal Learning Test (CVLT) immediate (List A) and Delayed Free Recall (DFR), the Benton Visual Retention Test (BVRT, # of errors), Brief Test of Attention (BTA), Animal Fluency test (AF), the Digit Span Forward and Backwards tests (DS-F and DS-B), the Clock Drawing Test (CDT), Trailmaking test parts A and B (TRAILS A and B, in seconds), (described in detail in Method S2). Cognitive domains spanned global mental status, verbal memory, verbal fluency, attention, visual memory, visuo-spatial abilities, and executive function, which includes working memory. A total of 11 cognitive test scores were computed from these tests. Total MMSE was normalized using previously described methods [23]; while Trails A and B scores (in seconds) were Log_e_ transformed to achieve pseudo-normality. With the exception of BVRT, Trails A and B, all test scores were in the direction of higher values reflecting better performance at v_1_ or over time.

### Plasma neurofilament light (NfL)

Fasting, morning plasma samples were collected into EDTA blood collection tubes. Tubes were centrifuged at 600×*g* for 15 min and the buffy coat was removed. These steps were repeated two times and the samples were visually examined for hemolysis. Plasma was aliquoted and stored at −80 °C until use. Plasma NfL levels were measured by Quanterix (Billerica, MA, USA) using the Simoa^®^ NF-light Advantage Kit following the kit instruction. Longitudinal samples for each person were run on the same plate and the proportion of people in each demographic group (race/sex/poverty) were balanced across all plates. Plasma samples were diluted 1:4 and concentrations reflect the dilution correction. Pooled plasma samples from two individuals were run in duplicate on all plates. These duplicate pooled plasma samples were used to calculate both the within plate (intra-assay) and between plates (inter-assay) coefficient of variation (CV). The average intra-assay CV was 4.5% and the average inter-assay CV was 7%. The analytical limit of detection (LOD) was calculated as 2.5 standard deviations above the background (mean of calibrator blanks). For the analytical lower limit of quantification (LLOQ), triplicate measurements of serially diluted calibrator were run as unknowns and read on the standard calibration curve. The LLOQ was determined as the lowest dilution with a pooled CV ≤ 20% and a sample read back recovery between 80 and 120% of the expected concentration. The analytical upper limit of detection (ULOQ) was the highest concentration of the calibrator curve. Analytical LOD, LLOQ, and ULOD values were converted to functional values by multiplying by the dilution factor (4×) to enable direct comparison to the sample results. The functional LOD and the functional LLOQ were 0.152 and 0.696 pg/ml, respectively. The functional ULOD was 1872 pg/ml.

### Covariates

Several covariates were considered in this study as potential confounders, given their previously shown association with cognitive performance or decline, which may also be associated with NfL exposures. These included v_1_ age (continuous, years), sex (male, female), race (White, African American), poverty status (below vs. above 125% the federal poverty line), educational attainment (less than high school, high school, more than high school), and literacy (Wide Range Achievement Test, third edition [WRAT-3]). Age at v_2_ was also used to compute time between v_1_ and v_2_, a measure relevant to our main models. Poverty status was operationalized using the 2004 US Census Bureau poverty thresholds [24] based on household income and total family size (including children <18 years). Furthermore, lifestyle and health-related factors were among those considered as potential confounders, given their potential impact on both exposures and outcomes. Those factors included current smoking status (0 = no vs. 1 = yes), illicit drug use (0 = no vs. 1 = yes, using any of marijuana, opiates, and cocaine), body mass index (BMI, weight/height^2^, kg m^−2^, continuous), self-rated health status categorized as 0=poor/average (referent), 1 = good and 2 = very good/excellent, the Healthy Eating Index 2010 (HEI-2010) [25], measuring overall diet quality based on food and macronutrient-related guidelines for Americans, total energy intake (kcal/d), and the 20-item CES-D total score for depressive symptoms. Moreover, an unweighted co-morbidity index was also accounted for. This index was composed of hypertension (0 = no, 1 = yes), diabetes (0 = diabetic, 1 = pre-diabetic, 2 = diabetic) and dyslipidemia (or statin use) (0 = no, 1 = yes), and self-reported history of any of several cardiovascular disease conditions (0 = no, 1 = yes). The latter component screened for the occurrence of several conditions, namely atrial fibrillation, angina, coronary artery disease, congestive heart failure, and myocardial infarction. Consequently, the co-morbidity index could potentially range between 0 and 5.

### Statistical methods

Stata release 16 [26] was used to conduct all analyses. We first described the analytic sample’s characteristics at baseline using means and proportions with bivariate linear, logistic, and multinomial logit models to examine racial differences in continuous, binary, and categorical multi-level covariates, respectively. We then adjusted those models for age, sex, and poverty status to determine whether racial differences remained statistically significant. Second, for testing our main hypotheses, a series of linear models were conducted (mixed-effects and ordinary least-square, OLS) (Method S3 for mixed-effects models). Separate analyses for 11 cognitive test scores were conducted, adjusting for two sets of covariates: Model 1: only socio-demographic variables: age at v1, sex, race, and poverty status; Model 2: socio-demographics + all other lifestyle and health-related covariates. To reduce missing data due to the addition of covariates into different models, given that each covariate had, individually <5% missing on average, we ensured sample sizes were constant between reduced and fully adjusted models by conducting multiple imputations (five imputations, ten iterations), using the chained equations methodology. All covariates were used simultaneously during this estimation process, similar to previous studies [27, 28] and continuous covariates were centered around their means. Thus, for mixed-effects linear regression models, we applied Models 1 and 2 to two exposures (NfL and δNfL), 11 cognitive test scores with up to two repeats (effect of exposures on v_1_ cognitive performance (CP_v1_) and cognitive performance change over time (δCP)), one main stratifying variable (race), and several exploratory stratifying variables (sex, age group, and poverty status). NfL was Log_e_ transformed in all these analyses, and the annualized changes in the Log_e_ transformed NfL between v_1_ and v_2_ were used to operationalize δNfL [i.e., δNfL = (Log_e_(NfL_v2_) − Log_e_(NfL_v1_)/(Age_v2_ − Age_v1_)], using complete case analysis. *Z*-scoring for exposures was done using the final eligible sample (*N* = 625). These two exposures were constructed in a similar way in other studies (e.g., [21]). Racial differences in the association between NfL exposures and cognitive performance at v_1_ was tested using NfL × Race and δNfL × Race interaction terms in separate models, while that of the association between NfL exposures and cognitive change was carried out by testing the NfL/δNfL × TIME × Race term in the same model. Following a similar approach but with a set of OLS linear regression models, race-specific associations of v_1_ NfL and δNfL with v_2_ cognitive performance (CP_v2_) as an outcome of interest, were examined, while additionally adjusting models with the time of follow-up (years) between v_1_ and v_2_. Racial differences were also tested using two-way interaction terms (NfL × Race) in unstratified models, as were differences by age group, sex, and poverty status.

In all models, sample selectivity due to missing exposure and outcome data, relative to the initially recruited sample, was adjusted for using a two-stage Heckman selection strategy. Thus, we first predicted an indicator of selection with socio-demographic factors, namely, v_1_ age, race, sex, and poverty status using a probit regression model, which yielded an inverse mills ratio (IMR), a function of the probability of being selected given those socio-demographic factors. At a second stage, we estimated our multiple mixed-effects and OLS linear regression models adjusted for the IMR in addition to the aforementioned covariates [29].

This study set the Type I error rate a priori for main and interactive effects before correction for multiple testing to 0.05 and 0.10, respectively [30]. We accounted for outcome multiplicity (i.e., 11 cognitive test scores) using the approach of familywise Bonferroni correction [31], specifically for Model 1. Subsequently, the full model (Model 2) was considered a sensitivity model in which potentially confounding and/or mediating factors were included. In addition, a reduced version of Model 2 (Model 3) was tested, whereby only covariates, aside from those included in Model 1, shown to be associated with each of the two exposures were included. This model was only conducted as a sensitivity analysis. Therefore, we adjusted significance levels for main effects to *p* < 0.00455 (0.05/11), and for two-way interaction terms to 0.10/11 = 0.00910, similar to previous work [32]. Moreover, *q*-values (false discovery rates) were also computed as an alternative means to correct for multiple testing in Model 1, accounting for multiplicity in cognitive tests only [33, 34]. *Q*-values < 0.05 were used for statistical significance for main effects (e.g., effect of NfL_v1_), while 0.05 ≤ *q*-values < 0.10 were considered as significant for two-way interaction terms (e.g., effect of NfL_v1_ × *TIME*). In our exploratory stratified analysis, all main hypotheses were tested across sex, age group (≤50 y, >50 y, as 50 y was the approximate median age) and poverty status (above vs. below poverty), separately, using the same modeling approach; and only familywise Bonferroni correction was applied to this part of the analysis (Model 1). Main findings were illustrated using predictive margins (with estimated 95% CI) of outcomes across time, and by exposure, overall or stratified by race and/or the other socio-demographic factors, using a specific mixed-effects or OLS linear regression model. Data analysis code in parts or in full can be made available upon request to the corresponding author.

## Results

Overall, and based on Table 1, participants were ~48 years old at initial testing; African Americans were significantly older than Whites (48.7 vs. 47.3, *p* < 0.05). A significantly higher proportion of Whites than African Americans had <HS education (7.9% vs. 3.7%). Although there were no race differences in poverty status, mean literacy was significantly higher among Whites. Log_e_ transformed NfL_v1_ plasma concentration was significantly higher among Whites compared with African Americans. However, there were no significant differences between races in the annualized rate of change values of NfL (delta NfL; δNfL). Current drug use was higher among African Americans; CES-D total score was higher among Whites. Although the co-morbidity index did not differ by race, dyslipidemia was more prevalent among Whites and hypertension was more prevalent among African Americans. In this select sample, Whites performed better than African Americans on most cognitive tests at v_1_. Whites had a greater rate of decline on CVLT-List A and a smaller rate of decline on the BVRT than African Americans.

**Table 1:** Study sample characteristics, overall and by race in the final analytic sample with imputed covariates (*N* = 625), HANDLS 2004–2013^a^.

	Overall	Whites	African American
	(*X* ± SE), %	(*X* ± SE), %	(*X* ± SE), %
	(*N* = 625)	(*N* = 273)	(*N* = 352)
*X* ± SE or %±SE			
NfL at v_1_, pg/mL			
Log_e_ transformed	+1.976 ± 0.020	+2.114 ± 0.029^****e^	+1.870 ± 0.026
Annualized rate of change in Log_e_ NfL between v_1_ and v_2_ δNfL	+0.044 ± 0.004	+0.038 ± 0.006	+0.050 ± 0.005
Baseline socio-demographic, SES and health-related variables			
Sex, % male	41.6 ± 2.0	40.3 ± 3.0	42.6 ± 2.6
Age at v_1_, yrs.	47.9 ± 0.36	48.7 ± 0.51^**^	47.3 ± 0.51
African American, %	56.3 ± 2.0	0.00	100.0
Poverty status, % <125% of the 2004 federal poverty guidelines	27.8 ± 1.8	26.0 ± 2.7	29.3 ± 2.4
Education, Completed, %			
<HS	5.5 ± 0.9	7.9 ± 1.6^**e^	3.7 ± 1.0
HS	57.3 ± 2.0	57.3 ± 3.1	57.4 ± 2.6
>HS	37.1 ± 2.0	34.8 ± 3.0	38.9 ± 2.6
Literacy, WRAT-3 score	43.1 ± 0.3	44.9 ± 0.5^****e^	41.6 ± 0.4
Baseline drug and tobacco use			
Any drug, current user, %	16.3 ± 1.6	13.0 ± 2.1^*^	18.9 ± 2.2
Tobacco, current user, %	40.0 ± 2.0	39.2 ± 3.0	40.6 ± 2.6
BMI, kg/m^2^	30.2 ± 0.3	30.1 ± 0.4	30.2 ± 0.4
Self-rated health, %			
Poor/Average,	19.4 ± 1.6	24.5 ± 2.6^***e^	15.3 ± 1.9
Good	41.3 ± 2.0	37.4 ± 2.9	44.3 ± 2.6
Very good/excellent	39.4 ± 2.0	38.1 ± 2.9	40.3 ± 2.6
HEI-2010 total score at v_1_	42.1 ± 0.6	41.2 ± 0.8^*e^	42.8 ± 0.6
Total energy intake, kcal/day	1,986 ± 44	1,995 ± 64	1,978 ± 56.1
CES-D total score	14.1 ± 0.4	15.1 ± 0.70^**e^	13.3 ± 0.57
Hypertension^b^, %	41.0 ± 2.0	36.2 ± 2.9^***e^	44.7 ± 2.7
Diabetes^b^, %			
No	66.3 ± 2.0	62.2 ± 3.0	69.5 ± 2.6
Pre-diabetic	21.7 ± 1.6	24.5 ± 2.6^*^	19.4 ± 2.2
Diabetic	12.0 ± 1.4	13.3 ± 2.1	11.1 ± 1.7
Dyslipidemia^b^, %	25.6 ± 1.8	29.4 ± 2.9^*^	22.7 ± 2.4
Cardiovascular disease^b^, %	13.2 ± 1.4	11.6 ± 2.0	14.3 ± 2.0
Co-morbidity index^b^	3.26 ± 0.05	3.31 ± 0.08	3.23 ± 0.07
Cognitive performance at v_1_, unadjusted^c^			
MMSE, normalized	76.9 ± 0.6	79.8 ± 0.9^****e^	74.6 ± 0.8
CVLT-List A	24.90 ± 0.28	26.1 ± 0.4^****e^	23.9 ± 0.4
CVLT-DFR	7.61 ± 0.13	8.30 ± 0.20^****e^	7.06 ± 0.17
BVRT	6.17 ± 0.20	5.93 ± 0.29	6.35 ± 0.27
BTA	6.80 ± 0.09	7.09 ± 0.14^***e^	6.52 ± 0.12
AF	19.07 ± 0.22	19.53 ± 0.34^*^	18.71 ± 0.28
DS-F	7.29 ± 0.09	7.58 ± 0.13^***e^	7.07 ± 0.11
DS-B	5.61 ± 0.08	6.03 ± 0.14^****e^	5.28 ± 0.10
CDT	8.77 ± 0.05	8.94 ± 0.07^***e^	8.64 ± 0.06
Log_e_ (TRAILS A)	3.44 ± 0.02	3.36 ± 0.02^****e^	3.51 ± 0.02
Log_e_(TRAILS B)	4.57 ± 0.03	4.37 ± 0.04^****e^	4.72 ± 0.04
Annualized change in cognitive performance estimated between v_1_ and v_2_, unadjusted^c^			
MMSE, normalized	−0.06 ± 0.13	+0.02 ± 0.23	−0.05 ± 0.16
CVLT-List A	−1.25 ± 0.06^┼^	−1.43 ± 0.10^┼,**e^	−1.14 ± 0.06^┼^
CVLT-DFR	−0.44 ± 0.03^┼^	−0.47 ± 0.04^┼^	−0.41 ± 0.03^┼^
BVRT	+0.49 ± 0.04^┼^	+0.33 ± 0.06^┼,****e^	+0.59 ± 0.06^┼^
BTA	−0.052 ± 0.021^┼^	−0.033 ± 0.030	−0.059 ± 0.026^┼^
AF	+0.075 ± 0.038^┼^	+0.058 ± 0.066	+0.091 ± 0.047
DS-F	+0.011 ± 0.015	+0.030 ± 0.026	+0.003 ± 0.019
DS-B	+0.024 ± 0.016	+0.055 ± 0.029	+0.009 ± 0.019
CDT	−0.015 ± 0.013	−0.028 ± 0.021	−0.004 ± 0.017
Log_e_ (TRAILS A)	−0.0003 ± 0.0032	+0.0037 ± 0.0042	−0.004 ± 0.005
Log_e_(TRAILS B)	+0.0156 ± 0.005^┼^	+0.0234 ± 0.008^┼^	+0.010 ± 0.007

Abbreviations: *AF* Animal Fluency; *BMI* body mass index; *BTA* Brief Test of Attention; *BVRT* Benton Visual Retention Test; *CDT* Clock Drawing Test; *CES-D* Center for Epidemiologic Studies-Depression; *CVLT-DFR* California Verbal Learning Test-Delayed Free Recall; *CVLT-List A* California Verbal Learning Test-List A; *DS-B* Digits Span-Backward; *DS-F* Digits Span-Forward; *HANDLS* Healthy Aging in Neighborhoods of Diversity across the Life Span; *HEI-2010* Healthy Eating Index, 2010 version; *HS* high school; *MMSE* Mini-Mental State Examination; *SE* standard error; *TRAILS A* Trailmaking test, part A; *TRAILS B* Trailmaking test, part B; *WRAT-3* Wide Range Achievement Test, 3rd revision; *X* mean.

*p < 0.10; **p < 0.05; *** *p* < 0.010; ****p < 0.001, *t*-test for the null hypothesis of no between-race differences.

^┼^*p* < 0.05, *t*-test for the null hypothesis of *γ*_1_ = 0 (fixed effects coefficient for *TIME*) in mixed-effects linear regression models with *TIME* as the only variable.

^a^Values are means (*X*) ± SE for continuous variables and % for categorical variables. The sample selected has complete data on MMSE and 10 other cognitive test scores at visits 1 and/or 2 and complete data on ApoE genotypes. Other covariates were multiple imputed (five imputations with ten iterations), using chained equations. All cognitive test scores are in the direction of higher score → better performance with the exception of BVRT (# of errors) and TRAILS A and B (# of sec. to complete).

^b^The co-morbidity index was calculated as the sum of hypertension, diabetes, and dyslipidemia (or statin use), and self-reported history of cardiovascular disease included atrial fibrillation, angina, coronary artery disease, congestive heart failure, or myocardial infarction, ranging from 0 to 5.

^c^Crude baseline cognitive test score. Sample sizes varied between 492 and 624 for the overall sample.

^d^Crude estimated the annual rate of change in cognitive performance based on mixed-effects linear regression model with TIME as the only covariate. Difference by race was determined by interacting TIME with race.

^e^*p* < 0.05 upon further adjustment for age, sex, and poverty status in multiple linear, logistic, multinomial logit, and mixed-effects linear regression models with race entered as the main predictor.

Our main hypotheses of associations between plasma NfL exposures and time-dependent cognitive outcomes were examined by mixed-effects and OLS regression models (Tables 2, 3) and are summarized in Fig. S1. Our exploratory analyses by age group, sex, and poverty status are presented in Tables S1−S3. Over a mean follow-up of 4.3 years, no association retained statistical significance upon correction for multiple testing in the total sample. However, we found initial NfL (i.e., NfL_v1_) was associated with faster decline on normalized mental status scores in Whites only (δMMSE_norm:_: *γ*_11_ = −0.661 ± 0.252, *P* = 0.0085, *q* = 0.094, reduced model), an association that retained significance in the fully adjusted model 2. This association (NfL_v1_ vs. decline in performance) was also found in those >50 years of age (δMMSE_norm_: *γ*_11_ = −0.705 ± 0.242, *P* = 0.004, reduced model); (Tables 2 and S2). Annualized increase in NfL was associated with greater decline in verbal fluency in men (δAF: *γ*_11_ = −0.181 ± 0.058, *P* = 0.002, full model); (Table S1). In other exploratory analyses (Tables S1−S3), annualized increase in NfL was associated with slower decline in verbal memory among individuals living above poverty (δCVLT-DFR: +0.104 ± 0.036, *P* = 0.004, reduced model), while, in the older group (>50 years), first-visit NfL was linked with better performance at baseline in global mental status and verbal memory (*P* < 0.004). Finally, and upon correction for multiple testing, no stratum-specific associations were found between NfL_v1_ (or δNfL) and follow-up cognitive performance. Reduction of Model 2 to Model 3, leaving in only additional covariates (in addition to socio-demographics) that were associated with NfL exposures, did not alter our main findings.

**Table 2:** Baseline and annual rates of change in plasma neurofilament light (v^1^ NfL, and δNfL) and their association with cognitive performance at v^1^ and change over time: overall and race-specific mixed-effects linear regression models: HANDLS 2004–2013^a^.

	NfL, pg/mL, (v_1_ Log_e_ transformed, *z*-scored)		δNfL, pg/mL (annualized change between v^1^ and v^2^, Log^e^ transformed, z-score)	
	Model 1	Model 2	Model 1	Model 2
	*γ* ± SE	*γ* ± SE	*γ* ± SE	*γ* ± SE
**Overall**	**(*N*** = **625,** ***k*** = 1.9−2.0)	**(*N*** = **625,** ***k*** = 1.9−2.0)	**(*N*** = **625,** ***k*** = 1.9−2.0)	**(*N*** = **625,** ***k*** = 1.9−2.0)
*Outcome* *=* *Cognitive performance test score*				
Normalized MMSE				
Exposure, *γ*_*0a*_	+1.024 ± 0.667^b^	+0.688 ± 0.607^b^	+0.719 ± 0.574	+1.009 ± 0.508**
Exposure × TIME, *γ*_*1a*_	−0.254 ± 0.158^c^	−0.208 ± 0.161^c^	−0.004 ± 0.144	−0.086 ± 0.143
CVLT-List A				
Exposure, *γ*_*0a*_	+0.404 ± 0.310	+0.486 ± 0.302	−0.208 ± 0.264	−0.177 ± 0.249
Exposure × TIME, *γ*_*1a*_	−0.078 ± 0.067	−0.051 ± 0.070	+0.061 ± 0.060	+0.035 ± 0.060
CVLT-DFR				
Exposure, *γ*_*0a*_	+0.164 ± 0.144	+0.226 ± 0.143	−0.127 ± 0.122	−0.139 ± 0.118
Exposure × TIME, *γ*_*1a*_	−0.038 ± 0.031	−0.029 ± 0.032	+0.051 ± 0.027*	+0.045 ± 0.028
BVRT				
Exposure, *γ*_*0a*_	−0.072 ± 0.223	−0.211 ± 0.216	−0.038 ± 0.192	−0.042 ± 0.181
Exposure × TIME, *γ*_*1a*_	+0.059 ± 0.048	+0.058 ± 0.051	+0.005 ± 0.044	+0.015 ± 0.044
BTA				
Exposure, *γ*_*0a*_	+0.164 ± 0.105	+0.100 ± 0.105	+0.073 ± 0.088	+0.102 ± 0.086
Exposure × TIME, *γ*_*1a*_	−0.036 ± 0.025	-0.037 ± 0.026	−0.013 ± 0.022	−0.016 ± 0.022
AF				
Exposure, *γ*_*0a*_	−0.107 ± 0.253	−0.176 ± 0.250	+0.094 ± 0.218	+0.097 ± 0.210
Exposure × TIME, *γ*_*1a*_	+0.007 ± 0.046	+0.006 ± 0.048	−0.071 ± 0.041*	−0.077 ± 0.042*
DS-F				
Exposure, *γ*_*0a*_	+0.104 ± 0.103	+0.046 ± 0.098	−0.021 ± 0.089	0.0077 ± 0.083
Exposure × TIME, *γ*_*1a*_	−0.015 ± 0.018	−0.018 ± 0.019	−0.010 ± 0.016	−0.011 ± 0.017
DS-B				
Exposure, *γ*_*0a*_	+0.056 ± 0.097^b^	+0.009 ± 0.091	+0.066 ± 0.084	+0.094 ± 0.076
Exposure × TIME, *γ*_*1a*_	−0.023 ± 0.020	−0.025 ± 0.020	+0.005 ± 0.018	+0.000 ± 0.018
CDT				
Exposure, *γ*_*0a*_	+0.057 ± 0.056^b^	+0.040 ± 0.057^b^	−0.005 ± 0.048	−0.005 ± 0.048
Exposure × TIME, *γ*_*1a*_	−0.011 ± 0.015^c^	−0.010 ± 0.016^c^	−0.003 ± 0.014 ^c^	+0.001 ± 0.014 ^c^
Log_e_(TRAILS A)				
Exposure, *γ*_*0a*_	+0.032 ± 0.016*	+0.025 ± 0.017	+0.013 ± 0.014	+0.014 ± 0.014
Exposure × TIME, *γ*_*1a*_	−0.001 ± 0.004	−0.001 ± 0.004	+0.002 ± 0.004	+0.0021 ± 0.004
Log_e_(TRAILS B)				
Exposure, *γ*_*0a*_	+0.026 ± 0.029	+0.023 ± 0.028	+0.014 ± 0.025	+0.010 ± 0.024
Exposure × TIME, *γ*_*1a*_	−0.004 ± 0.006	−0.004 ± 0.006	+0.001 ± 0.006	+0.002 ± 0.006
**Whites**	**(*N*** = **273,** ***k*** = 1.9−2.0)	**(*N*** = **273,** ***k*** = 1.9−2.0)	**(*N*** = **273,** ***k*** = 1.9−2.0)	**(*N*** = **273,** ***k*** = 1.9−2.0)
*Outcome* *=* *Cognitive performance test score*				
Normalized MMSE				
Exposure, *γ*_*0a*_	+2.334 ± 0.988**	+1.616 ± 0.836*	+0.279 ± 0.880	+0.581 ± 0.731
Exposure × TIME, *γ*_*1a*_	**−0.661** ± 0.252***	−0.565 ± 0.246**	+0.060 ± 0.239	+0.005 ± 0.230
CVLT-List A				
Exposure, *γ*_*0a*_	+0.311 ± 0.458	+0.194 ± 0.433	−0.518 ± 0.395	−0.369 ± 0.370
Exposure × TIME, *γ*_*1a*_	−0.025 ± 0.116	+0.026 ± 0.120	+0.099 ± 0.106	+0.071 ± 0.108
CVLT-DFR				
Exposure, *γ*_*0a*_	+0.238 ± 0.214	+0.190 ± 0.208	−0.299 ± 0.184	−0.290 ± 0.178
Exposure × TIME, *γ*_*1a*_	−0.032 ± 0.049	−0.008 ± 0.050	+0.092 ± 0.045**	+0.086 ± 0.045*
BVRT				
Exposure, *γ*_*0a*_	-0.217 ± 0.319	-0.206 ± 0.294	+0.147 ± 0.283	+0.008 ± 0.257
Exposure × TIME, *γ*_*1a*_	+0.076 ± 0.067	+0.066 ± 0.069	−0.058 ± 0.063	−0.027 ± 0.063
BTA				
Exposure, *γ*_*0a*_	+0.375 ± 0.160**	+0.273 ± 0.158*	−0.010 ± 0.135	+0.013 ± 0.130
Exposure × TIME, *γ*_*1a*_	−0.072 ± 0.040*	−0.055 ± 0.040	−0.002 ± 0.036	−0.003 ± 0.035
AF				
Exposure, *γ*_*0a*_	−0.282 ± 0.379	−0.442 ± 0.363	+0.048 ± 0.335	−0.004 ± 0.321
Exposure × TIME, *γ*_*1a*_	+0.004 ± 0.075	−0.005 ± 0.077	−0.075 ± 0.070	−0.063 ± 0.071
DS-F				
Exposure, *γ*_*0a*_	0.122 ± 0.154	+0.030 ± 0.136	+0.118 ± 0.136	+0.108 ± 0.118
Exposure × TIME, *γ*_*1a*_	−0.021 ± 0.030	−0.025 ± 0.030	+0.012 ± 0.028	+0.016 ± 0.028
DS-B				
Exposure, *γ*_*0a*_	+0.217 ± 0.153	+0.103 ± 0.137	+0.167 ± 0.136	+0.207 ± 0.119*
Exposure × TIME, *γ*_*1a*_	−0.048 ± 0.033	−0.044 ± 0.034	+0.031 ± 0.031	+0.022 ± 0.031
CDT				
Exposure, *γ*_*0a*_	−0.075 ± 0.079	−0.066 ± 0.078	+0.085 ± 0.070	+0.091 ± 0.068
Exposure × TIME, *γ*_*1a*_	+0.036 ± 0.023	+0.035 ± 0.024	−0.041 ± 0.022*	−0.043 ± 0.022*
Log_e_(TRAILS A)				
Exposure, *γ*_*0a*_	+0.034 ± 0.021	+0.038 ± 0.020*	+0.004 ± 0.019	+0.004 ± 0.018
Exposure × TIME, *γ*_*1a*_	−0.002 ± 0.005	−0.001 ± 0.005	+0.002 ± 0.004	+0.001 ± 0.005
Log_e_(TRAILS B)				
Exposure, *γ*_*0a*_	+0.000 ± 0.041	+0.018 ± 0.037	+0.029 ± 0.036	+0.019 ± 0.032
Exposure × TIME, *γ*_*1a*_	−0.002 ± 0.009	−0.004 ± 0.009	+0.002 ± 0.008	+0.002 ± 0.008
**African American**	**(*N*** = **352,** ***k*** = 1.9)	**(*N*** = **352,** ***k*** = 1.9)	**(*N*** = **352,** ***k*** = 1.9)	**(*N*** = **352,** ***k*** = 1.9)
*Outcome* *=* *Cognitive performance test score*				
Normalized MMSE				
Exposure, *γ*_*0a*_	−0.183 ± 0.910	−0.075 ± 0.870	+1.153 ± 0.756	+1.305 ± 0.705*
Exposure × TIME, *γ*_*1a*_	+0.047 ± 0.203	+0.025 ± 0.210	−0.053 ± 0.180	−0.102 ± 0.182
CVLT-List A				
Exposure, *γ*_*0a*_	+0.336 ± 0.413	+0.562 ± 0.412	+0.097 ± 0.343	+0.053 ± 0.332
Exposure × TIME, *γ*_*1a*_	−0.099 ± 0.080	−0.108 ± 0.084	+0.040 ± 0.068	+0.023 ± 0.070
CVLT-DFR				
Exposure, *γ*_*0a*_	+0.066 ± 0.193	+0.216 ± 0.197	+0.039 ± 0.160	−0.009 ± 0.158
Exposure × TIME, *γ*_*1a*_	−0.046 ± 0.040	−0.052 ± 0.042	+0.022 ± 0.034	+0.017 ± 0.035
BVRT				
Exposure, *γ*_*0a*_	+0.158 ± 0.317	−0.117 ± 0.314	−0.228 ± 0.264	−0.139 ± 0.255
Exposure × TIME, *γ*_*1a*_	+0.025 ± 0.069	+0.034 ± 0.072	+0.046 ± 0.060	+0.043 ± 0.061
BTA				
Exposure, *γ*_*0a*_	+0.020 ± 0.140	+0.005 ± 0.141	+0.157 ± 0.116	+0.173 ± 0.115
Exposure × TIME, *γ*_*1a*_	−0.023 ± 0.032	−0.036 ± 0.034	−0.025 ± 0.028	−0.022 ± 0.028
AF				
Exposure, *γ*_*0a*_	−0.110 ± 0.336	−0.062 ± 0.337	+0.193 ± 0.280	+0.182 ± 0.275
Exposure × TIME, *γ*_*1a*_	+0.007 ± 0.059	+0.016 ± 0.061	−0.075 ± 0.051	−0.098 ± 0.052*
DS-F				
Exposure, *γ*_*0a*_	+0.081 ± 0.139	+0.043 ± 0.137	−0.130 ± 0.116	−0.085 ± 0.112
Exposure × TIME, *γ*_*1a*_	−0.011 ± 0.024	−0.013 ± 0.025	−0.023 ± 0.021	−0.025 ± 0.021
DS-B				
Exposure, *γ*_*0a*_	−0.086 ± 0.126	−0.082 ± 0.118	−0.020 ± 0.103	−0.021 ± 0.096
Exposure × TIME, *γ*_*1a*_	−0.004 ± 0.024	−0.007 ± 0.025	−0.008 ± 0.021	−0.007 ± 0.022
CDT				
Exposure, *γ*_*0a*_	+0.156 ± 0.079*	+0.134 ± 0.081	−0.072 ± 0.066	−0.070 ± 0.066
Exposure × TIME, *γ*_*1a*_	−0.046 ± 0.021**	−0.043 ± 0.021**	+0.022 ± 0.0180	+0.026 ± 0.018
Log_e_(TRAILS A)				
Exposure, *γ*_*0a*_	+0.025 ± 0.025	+0.008 ± 0.025	+0.020 ± 0.021	+0.027 ± 0.020
Exposure × TIME, *γ*_*1a*_	−0.001 ± 0.006	−0.001 ± 0.006	+0.003 ± 0.005	+0.003 ± 0.005
Log_e_(TRAILS B)				
Exposure, *γ*_*0a*_	+0.039 ± 0.042	+0.019 ± 0.042	−0.001 ± 0.035	−0.001 ± 0.034
Exposure × TIME, *γ*_*1a*_	−0.004 ± 0.009	−0.003 ± 0.009	+0.001 ± 0.007	+0.003 ± 0.007

*Abbreviations:AF* Animal Fluency; *BTA* Brief Test of Attention; *BVRT* Benton Visual Retention Test; *CDT* Clock Drawing Test; *CES-D* Center for Epidemiologic Studies-Depression; *CVLT-DFR* California Verbal Learning Test-Delayed Free Recall; *CVLT-List A* California Verbal Learning Test-List A; *DS-B* Digits Span-Backward; *DS-F* Digits Span-Forward; *HANDLS* Healthy Aging in Neighborhoods of Diversity across the Life Span; *HEI-2010* Healthy Eating Index, 2010 version; *k* number of observations/participant; *MMSE* Mini-Mental State Examination; *SD* standard deviation; *SE* standard error; *NfL* neurofilament light; *TRAILS A* Trailmaking test, part A; *TRAILS B* Trailmaking test, part B; *WRAT-3* Wide Range Achievement Test, 3rd revision; *X* mean.

*p < 0.10; **p < 0.05; ***p < 0.010; ****p < 0.001, test for null hypothesis of *γ* = 0. Bolded values passed correction for multiple testing; underlined values passed *q* < 0.05 correction for multiple testing in Model 1.

^a^Models 1A.1−1K.2 included each of NfL (Log_e_ transformed, *z*-scored) or δNfL (annualized change in Log_e_ transformed NfL, *z*-scored), separately as the main predictor for v1 cognitive performance and cognitive change over time (11 test scores), using a series of mixed-effects linear regression models, carried out in the overall population, and stratified by race. These models adjusted only for age, sex, race, poverty status, and the inverse mills ratio. Models 2A.1−2K.2 followed a similar approach but adjusted further for selected socio-demographic, lifestyle, and health-related factors, namely educational attainment, the WRAT-3 score, current drug use, current tobacco use, body mass index, self-rated health, co-morbidity index, HEI-2010, total energy intake, and the CES-D total score. 1 SD of baseline Log_e_(NfL) is estimated at 0.51; mean = 1.98. dNfL values are annualized changes in Log_e_ transformed NfL between v_1_ and v_2_, *z*-scored. 1 SD of annualized change in Log_e_(NfL) is estimated at 0.101; mean = 0.044.

^b^*p* < 0.05 for Race × NfL/δNfL in models that are unstratified by race to which this three-way interaction was included.

^c^*p* < 0.05 for Race × NfL/δNfL × *TIME* in models that are unstratified by race to which this two-way interaction was included.

**Table 3:** Baseline plasma neurofilament light (v^1^ NfL and δNfL) and their association with cognitive performance at v^2^: overall and race-specific multiple ordinary least square linear regression models: HANDLS 2004−2013^a^.

	Whites		African Americans	
	Model 1	Model 2	Model 1	Model 2
	*β* ± SE	*β* ± SE	*β* ± SE	*β* ± SE
NfL, pg/mL, (v_1_ Log_e_ transformed, *z*-scored)				
*Outcome* *=* *cognitive performance test score*				
Normalized MMSE	*N* = 258	*N* = 258	*N* = 330	*N* = 330
	−0.622 ± 0.915	−0.852 ± 0.869	+0.134 ± 0.923	+0.048 ± 0.935
CVLT-List A	*N* = 272	*N* = 272	*N* = 349	*N* = 349
	+0.208 ± 0.515	+0.243 ± 0.521	−0.180 ± 0.442	−0.062 ± 0.447
CVLT-DFR	*N* = 272	*N* = 272	*N* = 349	*N* = 349
	+0.117 ± 0.226	+0.148 ± 0.230	−0.139 ± 0.189	−0.033 ± 0.193
BVRT	*N* = 273	*N* = 273	*N* = 350	*N* = 350
	+0.127 ± 0.330	+0.146 ± 0.318	+0.311 ± 0.316	+0.089 ± 0.310
BTA	*N* = 266	*N* = 266	*N* = 346	*N* = 346
	+0.072 ± 0.153	+0.015 ± 0.149	−0.091 ± 0.146	−0.163 ± 0.146
AF	*N* = 273	*N* = 273	*N* = 351	*N* = 351
	−0.289 ± 0.386	−0.488 ± 0.387	−0.111 ± 0.332	−0.032 ± 0.338
DS-F	*N* = 258	*N* = 258	*N* = 345	*N* = 345
	+0.041 ± 0.169	−0.073 ± 0.155	+0.012 ± 0.145	−0.039 ± 0.145
DS-B	*N* = 257	*N* = 257	*N* = 343	*N* = 343
	−0.037 ± 0.170	−0.160 ± 0.153	−0.103 ± 0.136	−0.123 ± 0.133
CDT	*N* = 273	*N* = 273	*N* = 351	*N* = 351
	+0.065 ± 0.088	+0.067 ± 0.090	−0.055 ± 0.080	−0.074 ± 0.083
Log_e_(TRAILS A)	*N* = 273	*N* = 273	*N* = 351	*N* = 351
	+0.024 ± 0.023	+0.031 ± 0.022	+0.021 ± 0.025	+0.007 ± 0.026
Log_e_(TRAILS B)	*N* = 272	*N* = 272	*N* = 351	*N* = 351
	−0.001 ± 0.043	+0.008 ± 0.04	+0.023 ± 0.046	+0.01 ± 0.045
δNfL, pg/mL (annualized change between v_1_ and v_2_, Log_e_ transformed, *z*-scored)				
*Outcome* *=* *Cognitive performance test score*				
Normalized MMSE	*N* = 258	*N* = 258	*N* = 330	*N* = 330
	+0.484 ± 0.812	+0.692 ± 0.756	+1.371 ± 0.780*	+1.144 ± 0.783
CVLT-List A	*N* = 272	*N* = 272	*N* = 349	*N* = 349
	−0.193 ± 0.457	−0.135 ± 0.456	+0.270 ± 0.368	+0.186 ± 0.365
CVLT-DFR	*N* = 272	*N* = 272	*N* = 349	*N* = 349
	+0.032 ± 0.201	+0.043 ± 0.202	+0.154 ± 0.157	+0.097 ± 0.157
BVRT	*N* = 273	*N* = 273	*N* = 350	*N* = 350
	−0.048 ± 0.293	−0.062 ± 0.272	−0.049 ± 0.265	+0.034 ± 0.253
BTA	*N* = 266	*N* = 266	*N* = 346	*N* = 346
	+0.007 ± 0.135	+0.016 ± 0.129	+0.054 ± 0.122	+0.073 ± 0.118
AF	*N* = 273	*N* = 273	*N* = 351	*N* = 351
	−0.217 ± 0.343	−0.234 ± 0.334	−0.141 ± 0.277	−0.211 ± 0.275
DS-F	*N* = 258	*N* = 258	*N* = 345	*N* = 345
	+0.168 ± 0.147^b^	+0.165 ± 0.132	−0.219 ± 0.120 *^b^	−0.186 ± 0.119
DS-B	*N* = 257	*N* = 257	*N* = 343	*N* = 343
	+0.296 ± 0.148**^b^	+0.315 ± 0.13**	−0.060 ± 0.115^b^	−0.067 ± 0.110
CDT	*N* = 273	*N* = 273	*N* = 351	*N* = 351
	−0.064 ± 0.078	−0.069 ± 0.078	+0.023 ± 0.067	+0.042 ± 0.068
Log_e_(TRAILS A)	*N* = 273	*N* = 273	*N* = 351	*N* = 351
	+0.012 ± 0.020	+0.013 ± 0.019	+0.033 ± 0.021	+0.037 ± 0.021*
Log_e_(TRAILS B)	*N* = 272	*N* = 272	*N* = 351	*N* = 351
	+0.033 ± 0.038	+0.031 ± 0.035	0.000 ± 0.038	+0.009 ± 0.037

*Abbreviations*: *AF* Animal Fluency; *BTA* Brief Test of Attention; *BVRT* Benton Visual Retention Test; *CDT* Clock Drawing Test; *CES-D* Center for Epidemiologic Studies-Depression; *CVLT-DFR* California Verbal Learning Test-Delayed Free Recall; *CVLT-List A* California Verbal Learning Test-List A; *DS-B* Digits Span-Backward; *DS-F* Digits Span-Forward; *HANDLS* Healthy Aging in Neighborhoods of Diversity across the Life Span; *HEI-2010* Healthy Eating Index, 2010 version; *MMSE* Mini-Mental State Examination; *k* number of observations/participant; *SD* standard deviation; *NfL* neurofilament light; *TRAILS A* Trailmaking test, part A; *TRAILS B* Trailmaking test, part B; *WRAT-3* Wide Range Achievement Test, 3rd revision.

*p < 0.10; **p < 0.05; ***p < 0.010; ****p < 0.001, test for null hypothesis of *β* = 0. Bolded values (if any) passed correction for multiple testing; Underlined values (if any) passed *q* < 0.05 correction for multiple testing in Model 1.

^a^Models 1A.1−1K.2 included each of NfL (Log_e_ transformed, *z*-scored) or δNfL (annualized change in Log_e_ transformed NfL, *z*-scored), separately as the main predictor for v2 cognitive performance (11 test scores), using a series of multiple linear regression models, stratified by race. These models adjusted only for age, sex, race, poverty status, length of follow-up (years), and the inverse mills ratio. Models 2A.1−2K.2 followed a similar approach but adjusted further for selected socio-demographic, lifestyle, and health-related factors, namely educational attainment, the WRAT-3 score, current drug use, current tobacco use, body mass index, self-rated health, co-morbidity index, HEI-2010, total energy intake, the CES-D total score. 1 SD of baseline Log_e_(NfL) is estimated at 0.51; mean = 1.98. dNfL values are annualized changes in Log_e_ transformed NfL between v_1_ and v_2_, *z*-scored. 1 SD of annualized change in Log_e_(NfL) is estimated at 0.101; mean = 0.044.

^b^*p* < 0.05 for Race × NfL in models that are unstratified by race to which this two-way interaction was included.

**Fig. 2:**
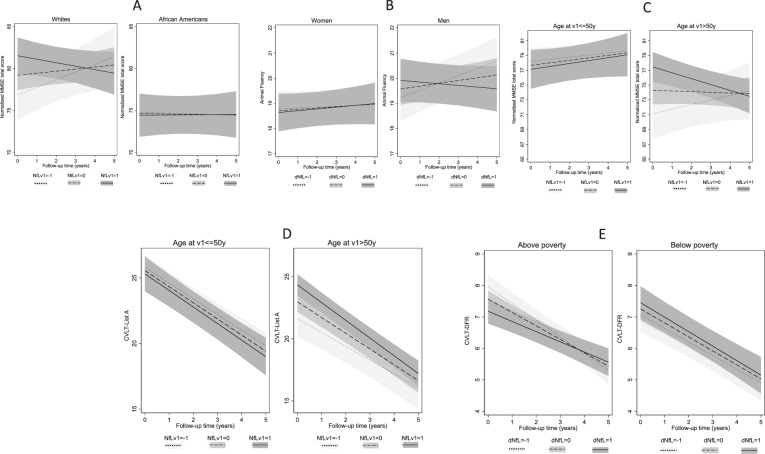
Summary of key findings by race, sex, age group, and poverty status across NfL exposures^a,b^. ^a^NfLv_1_ values are Log_e_ transformed and *z*-scored. Levels of exposure are −1: mean − 1 SD; 0: at mean; +1: mean + 1 SD. 1 SD of baseline Log_e_(NfL) is estimated at 0.51; mean = 1.98. dNfL values are annualized changes in Log_e_ transformed NfL between v_1_ and v_2_, z-scored. 1 SD of annualized change in Log_e_(NfL) is estimated at 0.101; mean = 0.044. All test scores presented in these figures are coded in the direction of higher score → better performance. ^b^**A** Predicted margins for normalized MMSE total score across NfLv1 are based on Model 1 among Whites and African Americans in Table 2; **B** predicted margins for animal fluency scores across dNfL are based on Model 2 among women and men in Table S1; **C** predicted margins for normalized MMSE total score across NfLv1 are based on Model 1 among ≤50 y vs. >50 y age groups in Table S2; **D** predicted margins for CVLT-List A across NfLv1 are based on Model 1 among ≤50 y vs. >50 y age groups in Table S2; **E** predicted margins for CVLT-DFR across dNfL are based on Model 2 among “above poverty” vs. “below poverty” groups in Table S3. Abbreviations: *AF* Animal Fluency; *BC* baseline cognitive performance; *CVLT-DFR* California Verbal Learning Test-Delayed Free Recall; *CVLT-List A* California Verbal Learning Test-List A; *dNfL* *z*-scores of annualized rates of change NfL, Loge transformed; *NfL*_*v1*_ plasma NfL levels, Log_e_ transformed, z-scored at v_1_.

The main finding among Whites, for NfL_v1_ vs. normalized MMSE scores across time is presented in terms of predictive margins of outcome per SD of exposure in Fig. 2A. The Figure indicates that among those with higher NfL_v1_ (i.e., v_1_ Log_e_ transformed plasma NfL, *z*-scored: mean + 1 SD), normalized MMSE score was on a decline over a period of 5 years as opposed to participants with NfL_v1_ at the mean or at mean − 1 SD, whose performance was improving over time, from an initial low level. This was not the case among African Americans. Figure S1 summarizes findings from Model 1, across race, for all regression analyses with 11 cognitive test scores, three types of outcomes, and two exposures. Figure 2B−E shows predictive margins of cognitive performance tests across exposure levels (NfL_v1_ and δNfL: *z*-score for annualized change in Log_e_ transformed plasma NfL between v_1_ and v_2_) and by sex, age group, and poverty status, highlighting the key exploratory findings.

## Discussion

### Main findings

This study is one of the few to examine plasma NfL baseline level (NfL_v1_) and its annualized rate of change over a 5 y follow-up (δNfL) and the longitudinal associations with cognitive performance in middle-aged adults over the same period of time. The study was specifically conducted among a bi-racial urban cohort of middle-aged men and women who were free from dementia at baseline. The sampling strategy allowed us to examine key tested associations across racial groups, and secondarily across sex, age, and poverty status groups. Cognitive performance was measured twice for most selected participants, reflecting global mental status and domains of verbal memory and fluency, visual memory and visuo-spatial abilities, attention, and executive functions. Over a mean follow-up of 4.3 years, we found initial NfL was associated with a faster decline on normalized mental status scores in Whites only and in those >50 years old. Annualized increase in NfL was associated with a greater decline in verbal fluency in men. In other exploratory analyses, annualized increase in NfL was associated with slower decline in verbal memory among individuals living above poverty, while, in the older group (>50 years), first-visit NfL was linked with better performance at baseline in global mental status and verbal memory.

### Previous studies and biological mechanisms

Currently, methods to diagnose and monitor neuropathology are based on various imaging modalities, which are expensive with limited availability. CSF biomarkers, including NfL, have also been utilized, but require invasive procedures. Therefore, non-invasive biomarkers of neurocognitive decline are needed to identify those individuals at risk for AD and other neurodegenerative diseases. Plasma NfL may be one such non-invasive biomarker. Recent technological advances indicate that NfL levels measured in the blood, i.e., plasma NfL, are associated with AD diagnosis and with various cognitive, imaging, and biochemical disease measures [1, 15, 35]. CSF NfL also was inversely associated with the clinical dementia rating scale, the Recognition Memory Test [9], and the cognitive sub-scale of an AD assessment battery [10]. Several studies have indicated that CSF NfL is elevated in the early stages of dementia and is a strong predictor for cognitive decline in Aβ positive individuals [36, 37], and in the general non-demented older adult population [22]. Given that Aβ positivity alone was not sufficient to predict symptoms of cognitive decline in AD, identifying additional markers of neurodegeneration that are downstream from Aβ accumulation has high utility for screening individuals in pre-symptomatic trials [9].

Given the high correlation between plasma and CSF NfL levels, and the invasiveness of acquiring CSF, plasma NfL may have greater overall utility as a screening tool. Several recent studies have shown that plasma NfL may accurately predict the estimated year of onset for dementia [38, 39]. In fact, several recent studies have shown that serum or plasma NfL are direct indicators of axonal degeneration based on neuroimaging markers, including gray and white matter pathology [21, 40, 41], and can act as a proxy for hypometabolism in AD-vulnerable brain regions, particularly in Aβ-positive individuals [42]. Generally, the demyelination of axons triggers inefficiency in energy utilization, dysfunction of the mitochondria, and oxidative stress accumulation, alterations that increase axonal fragmentation and result in neurodegeneration [43]. The spread of such pathology can occur at independent tract locations and their associated gray matter structures [44]. Since such axonal retraction does not often occur simultaneously, it is more likely that baseline plasma NfL rather than follow-up or change in NfL, is associated with change or follow-up outcome of neurodegeneration, as well as adverse cognitive performance outcomes [40, 45]. This is in line with our main findings.

Among older adults, several studies have indicated that plasma NfL is a good predictor for cognitive decline or impairment, independently of neuroimaging markers. One recent study found that individuals with AD or fronto-temporal dementia cases had higher plasma NfL compared to cognitively normal controls, with no differences detected for other neuropsychiatric disorders [46]. Upon adjustment for baseline hippocampal atrophy and memory scores, plasma NfL predicted greater cognitive decline among the cognitively impaired [46]. Another study among older adults suggested that a combination of markers (low plasma Aβ42/Aβ40 ratio and high plasma NfL level) was associated with a greater decline in cognitive performance over time [20]. These findings were recently corroborated by Mielke and colleagues who examined both plasma and CSF NfL in relation to cognitive and neuroimaging outcomes in a small sample of older adults (*N* = 79, median age: 76 y) participating in the Alzheimer’s Disease Neuro-imaging (ADNI) study. Their findings indicate that elevated baseline plasma NfL may adequately predict cognitive decline and brain imaging neurodegenerative measures, with comparable effect sizes to baseline CSF NfL [21]. Furthermore, Rajan and colleagues found that 1,327 older participants, plasma NfL > 25.5 pg/ml (determined 4–8 y prior to AD onset) was associated with 110% faster cognitive decline over 16 y of follow-up, as well as a faster decline in cortical thickness [18]. Similarly, He and colleagues found that among 452 older adults, a combination of elevated Aβ and plasma NfL was associated with faster decline on the MMSE compared with lower levels, even upon adjustment for APOE4 status [20]. Moreover, Nyberg and colleagues found that plasma NfL, while reflecting white matter alteration, may not be a good predictor for cognitive impairment or impending AD [19]. Most recently, Rübsamen et. al. (2021) evaluated associations between NfL and tau serum levels, neuropsychological functioning, and brain structure among a sample of 385 adults aged 65+ years enrolled in the Memory and Morbidity in Augsburg Elderly study [16]. The authors used linear regression models adjusted for age, sex, years of education, and comorbidities and reported a cross-sectional association between NfL serum levels and neuropsychological functioning which included standardized cognitive tests spanning the domains of short-term memory, cognitive speed, attention, and motor speed [16]. Furthermore, in a study by Khalil and colleagues (2020), the authors examined age-related changes in NfL serum levels and their associations with brain structure and functioning [17]. In a sample of 335 men and women drawn from the prospective and ongoing Austrian Stroke Prevention Family Study, the authors used backwards stepwise regression while considering comorbidities and observed that individuals with elevated and more variable NfL serum levels tended to show accelerated rates of neuronal injury which may be attributed to subclinical comorbid pathologies [17]. Moreover, the authors reported that baseline NfL serum levels were negatively associated with annualized changes in scores obtained from the Mini-Mental State Examination [17]. Taken together, these studies may suggest associations between NfL levels and changes in brain volume which may, in turn, influence neuropsychological functioning.

Our data in middle-aged adults is in agreement with other studies among older adults, indicating the utility of blood-based NfL as a non-invasive biomarker of cognitive decline, which may allow for disease monitoring. Few studies have examined longitudinal change in blood levels of NfL. In one study of AD, longitudinal plasma NfL levels increased in individuals with several baseline AD-disease measures [10]. Here, we examined longitudinal changes in plasma NfL in non-demented middle-aged adults. Therefore, we were able to assess baseline and rates of change of NfL in relation to longitudinal cognitive test performance across race and other socio-demographic variables (sex, age group, and poverty status). This is important given the limited information about the longitudinal changes in plasma NfL, especially in non-diseased cohorts. These associations we found, highlight the underlying neurodegeneration that occurs over time and suggests that baseline plasma NfL levels in Whites and in individuals >50 y may be valuable to predict those individuals who will cognitively decline faster than others. The lack of association between NfL and cognitive decline among African Americans may be due to less variability in NfL and limited change in cognitive performance over time within this racial group, especially among middle-aged adults, as compared with Whites and therefore a reduced statistical power to detect such an association. Among Whites, the only other cognitive performance test that was suggestive of an association between first-visit NfL and cognitive decline over time was BTA, reflecting attention, though this relationship did not survive correction for multiple testing (*γ*_11_ = −0.072 ± 0.040, *p* < 0.10, Model 1).

More generally, our study detected few associations between plasma NfL and cognitive decline compared with other studies, due to several possible reasons. First, our sample consisted of middle-aged adults, while most other studies were conducted among older adults aged over 60 y at baseline. This would result in a less steep decline in cognition over time in our sample compared to others of older mean age at baseline, which in turn would reduce the statistical power to detect an association between exposure and change in cognition over time, keeping exposure variability the same across samples. However, younger age also results in less variability in the plasma NfL exposures, further reducing statistical power. Second, our sample consisted of a diverse group of middle-aged adults, whereas most other studies recruited middle to upper-middle-class White older adults. This difference in age group, racial, and SES composition is expected to yield diverging findings between our study and those of others, mainly due to differing baseline exposure and outcome levels. Finally, we have adjusted for a large number of potential confounders, including body mass index, and cardio-metabolic risk factors, some of which were shown to be associated with plasma NfL in previous studies [47, 48]. We also accounted for literacy, depressive symptoms, and other important factors that most other studies have not controlled for.

### Strengths and limitations

Our study has several notable strengths. First, it is one of the largest longitudinal studies to examine plasma NfL levels in relation to cognition, using data from a community-based population, and the first to do so among middle-aged adults. In addition, plasma NfL was detected and quantified in non-demented individuals, which adds value to utilizing this biomarker as an early marker to monitor cognitive decline over time. Second, we had access to an extensive battery of cognitive tests that spanned the main domains of cognition, as well as measuring global mental status. Test scores had mostly two repeats, as did the main exposure of interest, plasma NfL. Third, the well-balanced sampling of HANDLS allowed for stratification of our analyses by race, sex, age group, and poverty status. Fourth, we used advanced statistical techniques, including mixed-effects linear regression models, multiple imputations, and 2-stage Heckman selection to test our key hypotheses, while reducing confounding and selection biases. The availability of two concurrent repeats of exposures and outcomes, allowed us to examine relationships in a detailed and bi-directional manner, though mainly focusing on the potential impact of NfL on cognition, rather than the reverse direction. Nevertheless, our study also has some limitations. First, our study sample was relatively young with a low mean NfL at baseline, when compared to previous studies that examined these questions in older adults. In addition, cognitive decline was limited in that age group, and was only evident above the age of 50 y. This may have reduced our ability to detect an association between NfL at v_1_ and change in cognitive function in the overall population. However, our results among Whites and the older group, suggest that NfL at v_1_ may be a predictor of decline in global mental status in middle-age in those groups who have a high performance on the MMSE at baseline and are prone to decline over a period of ~5 y.

## Conclusions

In summary, first-visit NfL was primarily associated with the global mental status decline among Whites, while exhibiting inconsistent relationships in some exploratory analyses. More comparable longitudinal studies are needed among middle-aged adults to determine the utility of plasma NfL both at baseline and as a marker of change over time in relationship to cognitive performance and decline.

## Disclaimer

The views expressed in this article are those of the authors and do not necessarily reflect the official policy or position of Fort Belvoir Community Hospital, the Defense Health Agency, Department of Defense, or U.S. Government. Reference to any commercial products within this publication does not create or imply any endorsement by Fort Belvoir Community Hospital, theDefense Health Agency, Department of Defense, or U.S. Government.

## Supplementary information

Supplementary material
