# Supplementary material for: Plasma neurofilament light as a potential biomarker for cognitive decline in a longitudinal study of middle-aged urban adults
Source: Transl Psychiatry. 2021 Aug 21;11:436. doi: 10.1038/s41398-021-01563-9 (PMC8380245; doi:10.1038/s41398-021-01563-9)
Supplement: Supplementary file 1 — Supplementary material [file 41398_2021_1563_MOESM1_ESM.docx]

**Plasma neurofilament light as a potential biomarker for cognitive decline in a longitudinal study of middle-aged urban adults**

**By May A. Beydoun et. al**

**SUPPLEMENTAL METHOD, TABLE AND FIGURE LEGEND**

**Method S1: NfL sample section**

**Method S2: Description of cognitive tests, literacy and the CES-D**

**Method S3: Mixed-effects regression models**

**Table S1:** **Summary of exploratory analysis findings by sex**

**Table S2: Summary of exploratory analysis findings by age group**

**Table S3: Summary of exploratory analysis findings by poverty status**

**Figure S1.** **Summary of main findings by race, Model 1**

**Method S1: NfL sample selection**

Plasma NfL was quantified in a sub-cohort of participants from HANDLS from visits v_1_ (2004-2009), v_2_ (2009-2013) and v_3_ (2013-2018), from which we extracted data from only v_1_ and v_2_ for our present study. This sub-sample included participants from the HANDLS SCAN, an ancillary neuroimaging sub-study, (n=238)^1^ This sub-study of the HANDLS cohort excluded participants with a history of dementia, stroke, transient ischemic attack, and carotid endarterectomy, MRI contraindications, terminal illness, HIV positivity or other neurological disorders ^1^. All HANDLS SCAN participants included in this sub-study had donated plasma samples at three different visits except for one participant that had samples from only 2 of 3 visits. In addition, we also included participants (n=463; 1389 samples) that donated plasma samples at v_1_, v_2_ and v_3_, who were HIV negative, had complete cognitive tests [Trailmaking test, part A (TRAILS A) and Digits Span-Forward (DS-F)[ at v_1_ and v_2_, Centers of Epidemiologic Studies-Depression (CES-D) scores at all 3 visits and with no history of HIV, stroke, transient ischemic attack, dementia, epilepsy, Parkinson’s disease or brain cancer. Participants (n=3) were also included who had plasma samples available from v_1_, v_2_ and v_3_, who also had genome wide DNA methylation data at v_1_^2-4^. These participants had the exclusions listed above. Thus, overall, N=694 HANDLS participants had plasma NfL data at v_1_ and N=709 at v_2_.

**Method S2: Description of cognitive tests, literacy and the CES-D**

*Mini-Mental State Examination (MMSE)*

The MMSE ^5^ is a cognitive screener that captures global cognitive functioning by briefly measuring orientation, concentration, immediate and short-term memory, language and constructional praxis. Scores range from 0 to 30. Higher scores suggest better cognitive function.

*California Verbal Learning Test (CVLT)*

The CVLT ^6^ is a verbal learning and memory test that includes a 16-item word list. A modified version of the CVLT was used with three, as opposed to five, learning trials. Cued recall was not administered. To capture verbal learning and memory, CVLT outcomes variables were total correct score for List A (learning) and List A long-delay free recall (memory). The learning score ranged from 0 to 48 and the memory score ranged from 0 to 16. Higher scores indicate better verbal learning and memory. A more comprehensive description of CVLT can be found elsewhere ^6^.

*Benton Visual Retention Test* (BVRT)

The BVRT ^7^ is a measure of nonverbal memory and visuo-constructional abilities. Administration A, Form D was used. A modified error scoring system based off the BVRT manual was used to guide two trained examiners in scoring the BVRT. Resolution of discrepancies in scoring were attempted by the two examiners, however, if a consensus could not be achieved, MKT, a research psychologist, provided the score. The outcome variable was total errors, with higher values indicating lower visual memory scores.

*Digit Span Forward and Backward (DS-F and DS-B)*

The Wechsler Adult Intelligence Scale, Revised^8^ Digit Span Forward and Backward primarily capture attention and working memory, a component of executive function. The tests were administered according to the manual’s instructions. The outcome variable was the total score, which was the total number of correct answers for each test.

*Category Fluency*

Category fluency^9,10^ is a measure of semantic verbal fluency, where participants are asked to generate as many animals as possible within a 60 second duration. Higher scores indicate better category fluency. The outcome variable was the total number of correctly generated words (i.e., words that were *not* intrusions and perseverations).

*Brief Test of Attention (BTA)*

For the BTA ^11^, a test of divided auditory attention, the examiner administered up to 10 trials of letters and numbers (4-18 items) that increased in length with each trial. Only the numbers portion of the test was administered. For each trial, participants were asked to disregard the number of letters read, while tracking how many numbers were recited. They were also told to keep their hands in fists to avoid finger counting. The outcome variable was the total number of correct trials.

*Trail Making Tests A and B (TRAILS A and B)*

The Trail Making Tests A and B^12^ primarily capture attention and executive functioning, respectively. The main executive function subdomain that TRAILS B captures is set-shifting and cognitive control. Both trials also measure visuo-motor scanning and processing speed. Participants were asked to draw a line between consecutive numbers (TRAILS A) and alternate between numbers and letters (TRAILS B) as quickly as they could. They were informed that they were being timed. The examiner pointed out errors that were then corrected by the participant. Errors were captured via increased time. Scores for TRAILS A and B reflected seconds to completion, where higher scores indicate poorer performance.

*Clock Drawing Test – Clock to Command (CDT)*

The Clock Drawing Test ^13^ is a measure of visuo-spatial abilities, that also captures elements of memory and executive function. Participants are instructed to draw a clock, put in all of the numbers, and set the hands to 10 minutes past 11. Performance is based off correct drawings of the clock face (0-2), numbers (0-4) and hands (0-4). Scores ranged from 0 to 10, with higher scores indicating better performance. Participants who did not score a perfect score on the command portion of the test were also asked to copy a clock with the hands set to 10 minutes after 11.

*Wide Range Achievement Test – 3^rd^ Edition: Word and Letter Reading Subtest (WRAT)*

The WRAT Word and Letter Reading Subtest ^14^ is a test of reading ability that is often used as a proxy for literacy and quality of education. Participants were instructed to correctly read a list of 50 words that increased in difficulty. If the first five words were not correctly pronounced, letter reading was also administered. Standard instructions were used with the tan form. The outcome variable used was the total number of correctly pronounced words.

*Center for Epidemiological Studies Depression Scale (CES-D)*

The CES-D ^15^ is a 20-item measure of depressive symptomatology. Participants are asked to consider the frequency and severity of their symptoms over the last week. Scores ranged from 0 to 60. Scores of >16 indicated significant depressive symptoms and scores of >20 indicated a clinically significant amount of depressive symptoms.

**Method S3**: Mixed-effects regression models

The main multiple mixed-effects regression models can be summarized as follows:

**Multi-level models** vs. **Composite models**

| **Eq.**  **1.1-1.4** |  |  |  |
| --- | --- | --- | --- |

Where Y_ij_ is the outcome (Each cognitive test score measured at v_1_ and/or v_2_) for each individual “i” and visit “j”; is the level-1 intercept for individual i; is the level-1 slope for individual i; is the level-2 intercept of the random intercept ; is the level-2 intercept of the slope ; is a vector of fixed covariates for each individual *i* that are used to predict level-1 intercepts and slopes, which can include socio-demographic variables among others. In this analysis, mixed-effects regression models included NfL and δNfL exposures (X_ij_) along with covariates (Z_ij_). and are level-2 disturbances; is the within-person level-1 disturbance ^16^.

It is worth noting that the models were fit using the entire HANDLS cohort with complete data on either v1 or v2 on cognitive tests was used to improve reliability of predicted estimates.

**Supplemental References**

1 Waldstein, S. R. *et al.* Differential Associations of Socioeconomic Status With Global Brain Volumes and White Matter Lesions in African American and White Adults: the HANDLS SCAN Study. *Psychosom Med* **79**, 327-335, doi:10.1097/PSY.0000000000000408 (2017).

2 Beydoun, M. A. *et al.* Association between epigenetic age acceleration and depressive symptoms in a prospective cohort study of urban-dwelling adults. *J Affect Disord* **257**, 64-73, doi:10.1016/j.jad.2019.06.032 (2019).

3 Beydoun, M. A. *et al.* Accelerated epigenetic age and cognitive decline among urban-dwelling adults. *Neurology* **94**, e613-e625, doi:10.1212/WNL.0000000000008756 (2020).

4 Tajuddin, S. M. *et al.* Novel age-associated DNA methylation changes and epigenetic age acceleration in middle-aged African Americans and whites. *Clin Epigenetics* **11**, 119, doi:10.1186/s13148-019-0722-1 (2019).

5 Folstein, M. F., Folstein, S. E. & McHugh, P. R. "Mini-mental state". A practical method for grading the cognitive state of patients for the clinician. *J Psychiatr Res* **12**, 189-198 (1975).

6 Delis, D. C., Freeland, J., Kramer, J. H. & Kaplan, E. Integrating clinical assessment with cognitive neuroscience: construct validation of the California Verbal Learning Test. *J Consult Clin Psychol* **56**, 123-130 (1988).

7 Benton, A. L. (The Psychological Corportation, New York, 1974).

8 Wechsler, D. WAIS-R manual., (The Psychological Corporation, Cleveland, 1981).

9 Morris, J. C. *et al.* The Consortium to Establish a Registry for Alzheimer's Disease (CERAD). Part I. Clinical and neuropsychological assessment of Alzheimer's disease. *Neurology* **39**, 1159-1165 (1989).

10 Morris, J. C., Mohs, R. C., Rogers, H., Fillenbaum, G. & Heyman, A. Consortium to establish a registry for Alzheimer's disease (CERAD) clinical and neuropsychological assessment of Alzheimer's disease. *Psychopharmacol Bull* **24**, 641-652 (1988).

11 Schretlen, D., Bobholz, J. H. & Brandt, J. Development and psychometric properties of the Brief Test of Attention. *Clinical Neuropsychologist* **10**, 80-89 (1996).

12 Reitan, R. *Trail Making Test: Manual for Administration and Scoring*. (Reitan Neuropsychological Laboratory, 1992).

13 Rouleau, I., Salmon, D. P., Butters, N., Kennedy, C. & McGuire, K. QUANTITATIVE AND QUALITATIVE ANALYSES OF CLOCK DRAWINGS IN ALZHEIMERS AND HUNTINGTONS-DISEASE. *Brain and Cognition* **18**, 70-87 (1992).

14 Wilkinson, G. S. *Wide Range Achievement Test–Revision 3*. (Jastak Association, 1993).

15 Nguyen, H. T., Kitner-Triolo, M., Evans, M. K. & Zonderman, A. B. Factorial invariance of the CES-D in low socioeconomic status African Americans compared with a nationally representative sample. *Psychiatry Res* **126**, 177-187, doi:10.1016/j.psychres.2004.02.004 (2004).

16 Blackwell, E., de Leon, C. F. & Miller, G. E. Applying mixed regression models to the analysis of repeated-measures data in psychosomatic medicine. *Psychosom Med* **68**, 870-878, doi:10.1097/01.psy.0000239144.91689.ca (2006).

**Table S1. Summary of exploratory analysis findings by sex^a^**

|  | **Annual rate of change**  ***γ_1a_*±SE** | |  | **Baseline performance**  ***γ_0a_*±SE** | |  | **Follow-up performance**  **β±SE** | |
| --- | --- | --- | --- | --- | --- | --- | --- | --- |
|  | **Women** | **Men** |  | **Women** | **Men** |  | **Women** | **Men** |
|  | **N=365, k=1.9-2.0** | **N=260, k=1.9-2.0** |  | **N=365, k=1.9-2.0** | **N=260, k=1.9-2.0** |  | **N=345-364** | **N=243-260** |
| ***MODEL 1*** |  |  |  |  |  |  |  |  |
| **V1 NFL** |  |  |  |  |  |  |  |  |
| Normalized MMSE | -0.102±0.209 | -0.363±0.244 |  | +0.362±0.906 | +1.613±0.991 |  | -0.212±0.870 | -0.119±0.973 |
| CVLT-List A | -0.058±0.102 | -0.059±0.085 |  | -0.058±0.465 | +0.810±0.404** |  | -0.346±0.507 | +0.480±0.423 |
| CVLT-DFR | -0.025±0.044 | -0.039±0.042 |  | +0.031±0.221 | +0.289±0.177 |  | -0.066±0.217 | +0.092±0.186 |
| BVRT | 0.057±0.070 | +0.040±0.066 |  | +0.123±0.308 | -0.215±0.327 |  | +0.377±0.317 | +0.062±0.325 |
| BTA | -0.044±0.036 | -0.026±0.036 |  | +0.267±0.153* | +0.050±0.144 |  | +0.072±0.148 | -0.094±0.149 |
| AF | -0.060±0.064 | +0.086±0.067 |  | +0.043±0.335 | -0.338±0.386 |  | -0.259±0.337 | +0.036±0.385 |
| DS-F | -0.006±0.027 | -0.019±0.025 |  | +0.055±0.140 | +0.163±0.153 |  | +0.028±0.147 | +0.063±0.163 |
| DS-B | -0.001±0.028 | -0.038±0.028 |  | -0.052±0.134 | +0.161±0.143 |  | -0.076±0.147 | -0.060±0.155 |
| CDT | +0.006±0.022 | -0.035±0.023 |  | -0.001±0.079 | +0.100±0.079 |  | +0.087±0.084 | -0.075±0.081 |
| Log_e_ (TRAILS A) | +0.000±0.010 | -0.008±0.006 |  | +0.005±0.042 | +0.060±0.024** |  | +0.021±0.022 | +0.026±0.026 |
| Log_e_(TRAILS B) | -0.250±0.196 | -0.006±0.007 |  | +1.427±0.771 | +0.039±0.041 |  | +0.007±0.045 | +0.016±0.043 |
|  |  |  |  |  |  |  |  |  |
| **δNFL** |  |  |  |  |  |  |  |  |
| Normalized MMSE | -0.25±0.196 | +0.191±0.217 |  | +1.427±0.771* | -0.036±0.864 |  | +0.600±0.750 | +1.193±0.856 |
| CVLT-List A | +0.057±0.090 | +0.058±0.075 |  | -0.009±0.386 | -0.408±0.355 |  | +0.191±0.433 | -0.126±0.370 |
| CVLT-DFR | +0.076±0.040* | +0.024±0.037 |  | -0.213±0.183 | -0.032±0.155 |  | +0.058±0.185 | +0.129±0.162 |
| BVRT | +0.032±0.065 | -0.015±0.058 |  | -0.287±0.263 | +0.227±0.284 |  | -0.151±0.272 | +0.111±0.284 |
| BTA | -0.042±0.032 | +0.009±0.031 |  | +0.085±0.127 | +0.060±0.122 |  | -0.052±0.126 | +0.117±0.130 |
| AF | +0.025±0.060 | **-0.156±0.057***** |  | -0.081±0.285 | +0.241±0.335 |  | -0.019±0.289 | -0.433±0.335 |
| DS-F | +0.002±0.025 | -0.025±0.021 |  | -0.011±0.119 | -0.009±0.134 |  | -0.004±0.127 | -0.089±0.139 |
| DS-B | +0.024±0.026 | -0.016±0.024 |  | +0.041±0.114 | +0.096±0.125 |  | +0.138±0.127 | +0.051±0.132 |
| CDT | -0.024±0.020 | +0.011±0.020 |  | -0.014±0.068 | +0.002±0.068 |  | -0.111±0.072 | +0.061±0.071 |
| Log_e_ (TRAILS A) | +0.005±0.005 | +0.000±0.006 |  | +0.015±0.019 | +0.010±0.021 |  | +0.032±0.019* | +0.014±0.023 |
| Log_e_(TRAILS B) | -0.002±0.009 | +0.006±0.006 |  | +0.021±0.036 | +0.000±0.035 |  | +0.007±0.039 | +0.025±0.038 |
|  |  |  |  |  |  |  |  |  |
| ***MODEL 2*** |  |  |  |  |  |  |  |  |
| **V1 NFL** |  |  |  |  |  |  |  |  |
| Normalized MMSE | -0.011±0.219 | -0.394±0.245 |  | -0.175±0.841^b^ | +1.639±0.900*^b^ |  | -0.238±0.863 | -0.378±0.973 |
| CVLT-List A | -0.020±0.110 | -0.024±0.088 |  | -0.123±0.456 | +0.963±0.398** |  | -0.287±0.523 | +0.639±0.429 |
| CVLT-DFR | +0.009±0.047 | -0.025±0.044 |  | -0.008±0.225 | +0.372±0.177** |  | +0.002±0.224 | +0.187±0.188 |
| BVRT | +0.066±0.074 | +0.019±0.068 |  | +0.023±0.307 | -0.448±0.302 |  | +0.294±0.320 | -0.198±0.303 |
| BTA | -0.038±0.038 | -0.013±0.037 |  | +0.135±0.155 | +0.027±0.143 |  | -0.047±0.150 | -0.095±0.145 |
| AF | -0.065±0.068 | +0.105±0.069 |  | -0.017±0.343 | -0.562±0.371 |  | -0.363±0.351 | -0.128±0.383 |
| DS-F | -0.012±0.029 | -0.007±0.026 |  | -0.031±0.138 | +0.175±0.142 |  | -0.088±0.150 | +0.128±0.153 |
| DS-B | -0.008±0.029 | -0.024±0.028 |  | -0.141±0.127 | +0.168±0.134 |  | -0.206±0.142 | -0.022±0.146 |
| CDT | +0.013±0.022 | -0.027±0.023 |  | -0.017±0.082 | +0.084±0.080 |  | +0.048±0.088 | -0.074±0.085 |
| Log_e_ (TRAILS A) | +0.007±0.005 | -0.011±0.006* |  | -0.006±0.023 | +0.058±0.024** |  | +0.021±0.023 | +0.011±0.026 |
| Log_e_(TRAILS B) | +0.000±0.010 | -0.006±0.007 |  | -0.004±0.042 | +0.027±0.037 |  | +0.000±0.046 | +0.012±0.041 |
|  |  |  |  |  |  |  |  |  |
| **δNFL** |  |  |  |  |  |  |  |  |
| Normalized MMSE | -0.365±0.197* | +0.162±0.213 |  | +1.600±0.684** | +0.186±0.767 |  | +0.312±0.717 | +1.189±0.833 |
| CVLT-List A | +0.027±0.093 | +0.037±0.075 |  | +0.037±0.356 | -0.410±0.346 |  | +0.129±0.426 | -0.209±0.368 |
| CVLT-DFR | +0.069±0.040* | +0.017±0.038 |  | -0.224±0.175 | -0.043±0.152 |  | +0.029±0.183 | +0.094±0.162 |
| BVRT | +0.036±0.067 | -0.002±0.058 |  | -0.280±0.251 | +0.228±0.259 |  | -0.105±0.259 | +0.206±0.259 |
| BTA | -0.050±0.033 | +0.010±0.031 |  | +0.127±0.123 | +0.061±0.119 |  | -0.025±0.122 | +0.102±0.123 |
| AF | +0.021±0.061 | -0.181±0.058*** |  | -0.076±0.280 | +0.377±0.316 |  | -0.018±0.288 | -0.402±0.325 |
| DS-F | -0.004±0.026 | -0.030±0.021 |  | +0.009±0.113 | +0.020±0.122 |  | -0.001±0.122 | -0.086±0.127 |
| DS-B | +0.021±0.026 | -0.026±0.024 |  | +0.052±0.104 | +0.134±0.115 |  | +0.138±0.118 | +0.057±0.121 |
| CDT | -0.016±0.020 | +0.006±0.020 |  | -0.026±0.067 | +0.023±0.069 |  | -0.092±0.071 | +0.062±0.073 |
| Log_e_ (TRAILS A) | +0.005±0.005 | +0.000±0.006 |  | +0.021±0.019 | +0.004±0.021 |  | +0.035±0.019* | +0.014±0.022 |
| Log_e_(TRAILS B) | +0.001±0.009 | +0.006±0.006 |  | +0.025±0.034 | -0.009±0.032 |  | +0.016±0.037 | +0.026±0.035 |

*Abbreviations:* AF=Animal Fluency; BTA=Brief Test of Attention; BVRT=Benton Visual Retention Test; CDT=Clock Drawing Test; CES-D=Center for Epidemiologic Studies-Depression; CVLT-DFR=California Verbal Learning Test-Delayed Free Recall; CVLT-List A=California Verbal Learning Test-List A; DS-B=Digits Span-Backward; DS-F=Digits Span-Forward; HEI-2010=Healthy Eating Index, 2010 version; MMSE=Mini-Mental State Examination; k=number of observations/participant; SD=Standard Deviation; NfL=Neurofilament Light; TRAILS A=Trailmaking Test, Part A; TRAILS B=Trailmaking Test, Part B; WRAT-3 = Wide Range Achievement Test, 3rd revision; X = mean.

^a^ Models 1A.1-1K.2 included each of NfL (Log_e_ transformed, z-scored) or δNfL (annualized change in Log_e_ transformed NfL, z-scored), separately as the main predictor for v1 cognitive performance, cognitive change over time, and v2 cognitive performance (11 test scores), using a series of multiple linear mixed-effects and ordinary least square regression models, stratified by sex. These models adjusted only for age, sex, race, poverty status, length of follow-up (years) (for models with follow-up outcome) and the inverse mills ratio. Models 2A.1-2K.2 followed a similar approach but adjusted further for selected socio-demographic, lifestyle and health-related factors, namely educational attainment, the WRAT-3 score, current drug use, current tobacco use, body mass index, self-rated health, co-morbidity index, HEI-2010, total energy intake, and the CES-D total score. 1 SD of baseline Log_e_(NfL) is estimated at 0.51; Mean=1.98. dNfL values are annualized changes in Log_e_ transformed NfL between v_1_ and v_2_, z-scored. 1 SD of annualized change in Log_e_(NfL) is estimated at 0.101 ; Mean=0.044.

^b^ p<0.05 for Sex×NfL in models that are unstratified by sex to which this 2-way interaction was included.

**p* < 0.10** *p* < 0.05; *** *p* < 0.010; *****p*<0.001, test for null hypothesis of γ=0 or β=0. Bolded values passed correction for multiple testing in Model 1.

**Table S2. Summary of exploratory analysis findings by age group^a^**

|  | **Annual rate of change**  ***γ_1a_*±SE** | |  | **Baseline performance**  ***γ_0a_*±SE** | |  | **Follow-up performance**  **β±SE** | |
| --- | --- | --- | --- | --- | --- | --- | --- | --- |
|  | **≤50y** | **>50y** |  | **≤50y** | **>50y** |  | **≤50y** | **>50y** | |
|  | **N=353, k=1.9-2.0** | **N=268, k=1.9-2.0** |  | **N=353, k=1.9-2.0** | **N=268, k=1.9-2.0** |  | **N=337-355** | **N=251-269** | |
| ***MODEL 1*** |  |  |  |  |  |  |  |  | |
| **V1 NFL** |  |  |  |  |  |  |  |  | |
| Normalized MMSE | +0.053±0.202^b^ | **-0.705±0.242***^b^** |  | -0.532±0.858^b^ | **+3.158±1.04***^b^** |  | -0.194±0.846 | -0.293±0.998 | |
| CVLT-List A | -0.034±0.090 | -0.159±0.100 |  | -0.252±0.431^b^ | **+1.359±0.438***^b^** |  | -0.407±0.450 | 0.644±0.503 | |
| CVLT-DFR | +0.003±0.043 | -0.095±0.042** |  | -0.179±0.200^b^ | +0.607±0.205***^b^ |  | -0.135±0.197 | 0.179±0.212 | |
| BVRT | +0.033±0.059 | +0.075±0.082 |  | +0.233±0.275* | -0.504±0.366** |  | +0.385±0.292 | -0.075±0.359 | |
| BTA | -0.012±0.033 | -0.067±0.039 |  | +0.032±0.134 | +0.345±0.166 |  | -0.027±0.140 | 0.025±0.158 | |
| AF | +0.026±0.061 | -0.019±0.069 |  | -0.216±0.339 | +0.059±0.377 |  | -0.097±0.342 | -0.069±0.379 | |
| DS-F | -0.027±0.025 | +0.002±0.027 |  | +0.140±0.139 | +0.071±0.152 |  | -0.01±0.147 | 0.091±0.162 | |
| DS-B | -0.030±0.025 | -0.022±0.031 |  | -0.029±0.133 | +0.197±0.140 |  | -0.182±0.140 | 0.067±0.162 | |
| CDT | -0.024±0.020 | +0.005±0.025 |  | +0.067±0.072 | +0.046±0.086 |  | -0.035±0.072 | 0.073±0.098 | |
| Log_e_ (TRAILS A) | -0.036±0.039 | -0.003±0.006 |  | +0.018±0.019 | +0.044±0.027* |  | +0.021±0.023 | 0.028±0.024 | |
| Log_e_(TRAILS B) | -0.008±0.008 | +0.003±0.010 |  | +0.065±0.037* | -0.038±0.047 |  | +0.037±0.039 | -0.024±0.051 | |
|  |  |  |  |  |  |  |  |  | |
| **δNFL** |  |  |  |  |  |  |  |  | |
| Normalized MMSE | -0.145±0.182 | +0.168±0.235 |  | +0.737±0.735 | +0.426±0.917 |  | +0.503±0.738 | +1.178±0.876 | |
| CVLT-List A | +0.026±0.078 | +0.097±0.093 |  | -0.111±0.361 | -0.381±0.389 |  | -0.006±0.387 | +0.007±0.443 | |
| CVLT-DFR | +0.024±0.037 | +0.071±0.040* |  | -0.110±0.168 | -0.149±0.181 |  | +0.021±0.169 | +0.145±0.186 | |
| BVRT | -0.024±0.052 | +0.063±0.077 |  | +0.040±0.236 | -0.119±0.321 |  | -0.042±0.252 | +0.056±0.315 | |
| BTA | -0.029±0.028 | +0.002±0.036 |  | +0.146±0.110 | -0.035±0.146 |  | +0.033±0.121 | +0.016±0.139 | |
| AF | -0.040±0.054^b^ | -0.148±0.064**^b^ |  | -0.398±0.289 | +0.802±0.328** |  | -0.572±0.292* | +0.204±0.333 | |
| DS-F | -0.031±0.022 | +0.014±0.025 |  | +0.037±0.119 | -0.094±0.133 |  | -0.074±0.126 | -0.032±0.140 | |
| DS-B | 0.000±0.022 | +0.008±0.029 |  | +0.131±0.114 | -0.044±0.123 |  | +0.173±0.120 | -0.018±0.141 | |
| CDT | -0.005±0.018 | -0.006±0.023 |  | -0.001±0.061 | -0.017±0.075 |  | -0.016±0.062 | -0.041±0.086 | |
| Log_e_ (TRAILS A) | +0.006±0.004 | -0.001±0.006 |  | +0.005±0.017 | +0.021±0.023 |  | +0.025±0.020 | +0.018±0.021 | |
| Log_e_(TRAILS B) | 0.000±0.007 | +0.005±0.009 |  | -0.005±0.032 | +0.038±0.042 |  | -0.012±0.034 | +0.055±0.045 | |
|  |  |  |  |  |  |  |  |  | |
| ***MODEL 2*** |  |  |  |  |  |  |  |  | |
| **V1 NFL** |  |  |  |  |  |  |  |  | |
| Normalized MMSE | +0.087±0.206^b^ | -0.578±0.246*^b^ |  | -0.860±0.802^b^ | +2.784±0.915***^b^ |  | -0.401±0.840 | -0.109±0.982 | |
| CVLT-List A | -0.052±0.095 | -0.067±0.105 |  | -0.026±0.423^b^ | +1.245±0.436***^b^ |  | -0.326±0.451 | +0.878±0.519* | |
| CVLT-DFR | -0.004±0.045 | -0.053±0.045 |  | -0.114±0.202^b^ | +0.670±0.207**^b^ |  | -0.108±0.199 | +0.394±0.218* | |
| BVRT | +0.015±0.061 | +0.100±0.086 |  | +0.176±0.266 | -0.755±0.358** |  | +0.279±0.278 | -0.233±0.359 | |
| BTA | -0.005±0.034 | -0.069±0.039* |  | -0.059±0.135 | +0.287±0.162* |  | -0.110±0.139 | -0.034±0.156 | |
| AF | +0.027±0.063 | -0.021±0.073 |  | -0.280±0.322 | +0.040±0.385 |  | -0.171±0.335 | -0.077±0.397 | |
| DS-F | -0.037±0.026 | +0.002±0.029 |  | +0.100±0.133 | +0.031±0.146 |  | -0.102±0.140 | +0.053±0.164 | |
| DS-B | -0.041±0.026 | -0.020±0.033 |  | -0.045±0.125 | +0.158±0.132 |  | -0.257±0.132* | +0.013±0.157 | |
| CDT | -0.022±0.020 | -0.005±0.026 |  | +0.040±0.073 | +0.096±0.089 |  | -0.057±0.074 | +0.083±0.102 | |
| Log_e_ (TRAILS A) | 0.000±0.005 | -0.001±0.007 |  | +0.012±0.019 | +0.039±0.027 |  | +0.017±0.024 | +0.027±0.025 | |
| Log_e_(TRAILS B) | -0.008±0.008 | +0.002±0.010 |  | +0.061±0.035* | -0.042±0.047 |  | +0.036±0.038 | -0.034±0.051 | |
|  |  |  |  |  |  |  |  |  | |
| **δNFL** |  |  |  |  |  |  |  |  | |
| Normalized MMSE | -0.289±0.181 | +0.168±0.229 |  | +1.53±0.667* | +0.068±0.791 |  | +0.521±0.72 | +0.798±0.841 | |
| CVLT-List A | +0.021±0.080 | +0.081±0.094 |  | +0.046±0.346 | -0.51±0.378 |  | +0.130±0.380 | -0.187±0.449 | |
| CVLT-DFR | +0.026±0.038 | +0.064±0.040 |  | -0.054±0.163 | -0.226±0.177 |  | +0.067±0.167 | +0.052±0.188 | |
| BVRT | -0.017±0.052 | +0.038±0.080 |  | -0.061±0.221 | +0.042±0.308 |  | -0.106±0.231 | +0.201±0.310 | |
| BTA | -0.029±0.028 | +0.015±0.036 |  | +0.202±0.107* | -0.070±0.140 |  | +0.101±0.117 | -0.019±0.134 | |
| AF | -0.054±0.055 | -0.148±0.066** |  | -0.251±0.268^b^ | +0.805±0.328**^b^ |  | -0.518±0.278* | +0.178±0.340 | |
| DS-F | -0.027±0.022 | +0.010±0.027 |  | +0.120±0.110 | -0.155±0.126 |  | +0.030±0.117 | -0.107±0.138 | |
| DS-B | +0.002±0.023 | +0.004±0.030 |  | +0.209±0.103**^b^ | -0.106±0.114^b^ |  | +0.259±0.111**^b^ | -0.102±0.133^b^ | |
| CDT | -0.004±0.018 | -0.001±0.024 |  | +0.029±0.061 | -0.054±0.076 |  | +0.019±0.063 | -0.063±0.088 | |
| Log_e_ (TRAILS A) | +0.006±0.004 | -0.001±0.006 |  | +0.004±0.016 | +0.022±0.023 |  | +0.023±0.020 | +0.026±0.022 | |
| Log_e_(TRAILS B) | +0.001±0.007 | +0.004±0.009 |  | -0.021±0.029 | +0.054±0.041 |  | -0.019±0.032 | +0.076±0.043* | |

*Abbreviations:* AF=Animal Fluency; BTA=Brief Test of Attention; BVRT=Benton Visual Retention Test; CDT=Clock Drawing Test; CES-D=Center for Epidemiologic Studies-Depression; CVLT-DFR=California Verbal Learning Test-Delayed Free Recall; CVLT-List A=California Verbal Learning Test-List A; DS-B=Digits Span-Backward; DS-F=Digits Span-Forward; HEI-2010=Healthy Eating Index, 2010 version; MMSE=Mini-Mental State Examination; k=number of observations/participant; SD=Standard Deviation; SE=Standard Error; NfL=Neurofilament Light; TRAILS A=Trailmaking Test, Part A; TRAILS B=Trailmaking Test, Part B; WRAT-3 = Wide Range Achievement Test, 3rd revision; X = mean.

^a^ Models 1A.1-1K.2 included each of NfL (Log_e_ transformed, z-scored) or δNfL (annualized change in Log_e_ transformed NfL, z-scored), separately as the main predictor for v1 cognitive performance, cognitive change over time, and v2 cognitive performance (11 test scores), using a series of multiple linear mixed-effects and ordinary least square regression models, stratified by age group. These models adjusted only for age, sex, race, poverty status, length of follow-up (years) (for models with follow-up outcome) and the inverse mills ratio. Models 2A.1-2K.2 followed a similar approach but adjusted further for selected socio-demographic, lifestyle and health-related factors, namely educational attainment, the WRAT-3 score, current drug use, current tobacco use, body mass index, self-rated health, co-morbidity index, HEI-2010, total energy intake, and the CES-D total score. 1 SD of baseline Log_e_(NfL) is estimated at 0.51; Mean=1.98. dNfL values are annualized changes in Log_e_ transformed NfL between v_1_ and v_2_, z-scored. 1 SD of annualized change in Log_e_(NfL) is estimated at 0.101 ; Mean=0.044.

^b^ p<0.05 for Age(group)×NfL in models that are unstratified by race to which this 2-way interaction was included.

***p* < 0.10** *p* < 0.05; *** *p* < 0.010; *****p*<0.001, test for null hypothesis of γ=0 or β=0. Bolded values passed correction for multiple testing in Model 1.

**Table S3. Summary of exploratory analysis findings by poverty status group^a^**

|  | **Annual rate of change**  ***γ_1a_*±SE** | |  | **Baseline performance**  ***γ_0a_*±SE** | |  | **Follow-up performance**  **β±SE** | |
| --- | --- | --- | --- | --- | --- | --- | --- | --- |
|  | **Above Poverty** | **Below Poverty** |  | **Above Poverty** | **Below Poverty** |  | **Above Poverty** | **Below**  **Poverty** |
|  | **N=451, k=1.9-2.0** | **N=174, k=1.9-2.0** |  | **N=451, k=1.9-2.0** | **N=174, k=1.9-2.0** |  | **N=425-451** | **N=163-173** |
| ***MODEL 1*** |  |  |  |  |  |  |  |  |
| **V1 NFL** |  |  |  |  |  |  |  |  |
| Normalized MMSE | -0.144±0.199 | -0.401±0.261 |  | +0.969±0.823 | +0.983±1.124 |  | +0.356±0.796 | -1.824±1.093* |
| CVLT-List A | -0.086±0.086 | -0.049±0.102 |  | +0.357±0.369 | +0.335±0.561 |  | -0.005±0.408 | -0.153±0.576 |
| CVLT-DFR | -0.063±0.039 | +0.033±0.050 |  | +0.201±0.177 | -0.024±0.240 |  | -0.048±0.178 | +0.035±0.248 |
| BVRT | +0.084±0.061 | +0.011±0.081 |  | -0.196±0.259 | +0.27±0.422 |  | +0.167±0.273 | +0.563±0.411 |
| BTA | -0.007±0.031 | -0.084±0.043* |  | +0.103±0.123 | +0.261±0.205 |  | +0.051±0.124 | -0.185±0.200 |
| AF | -0.027±0.059 | +0.081±0.073 |  | -0.169±0.309 | -0.121±0.425 |  | -0.33±0.310 | +0.148±0.429 |
| DS-F | +0.000±0.022 | -0.028±0.032 |  | +0.128±0.123 | +0.020±0.188 |  | +0.133±0.132 | -0.164±0.195 |
| DS-B | -0.001±0.025 | -0.065±0.032** |  | -0.008±0.120 | +0.217±0.165 |  | -0.048±0.129 | -0.121±0.187 |
| CDT | +0.002±0.019 | -0.035±0.026 |  | +0.026±0.069 | +0.093±0.098 |  | +0.039±0.071 | -0.051±0.104 |
| Log_e_ (TRAILS A) | -0.001±0.004 | -0.003±0.008 |  | +0.031±0.018* | +0.029±0.034 |  | +0.028±0.019 | +0.021±0.034 |
| Log_e_(TRAILS B) | +0.002±0.007 | -0.011±0.012 |  | +0.020±0.035 | +0.028±0.055 |  | +0.030±0.036 | -0.029±0.063 |
|  |  |  |  |  |  |  |  |  |
| **δNFL** |  |  |  |  |  |  |  |  |
| Normalized MMSE | 0.000±0.193 | -0.013±0.222 |  | +0.608±0.712 | +0.939±0.961 |  | +1.113±0.706 | +0.778±0.905 |
| CVLT-List A | +0.099±0.080 | 0.000±0.084 |  | -0.559±0.318*^b^ | +0.660±0.462^b^ |  | -0.198±0.355 | +0.724±0.479 |
| CVLT-DFR | **+0.104±0.036***^b^** | -0.025±0.041^b^ |  | -0.330±0.152**^b^ | 0.290±0.197^b^ |  | +0.074±0.154 | +0.197±0.207 |
| BVRT | +0.045±0.057 | -0.053±0.070 |  | -0.020±0.224 | -0.183±0.361 |  | +0.139±0.238 | -0.487±0.346 |
| BTA | -0.012±0.028 | -0.011±0.036 |  | +0.073±0.105 | +0.051±0.166 |  | +0.053±0.107 | +0.005±0.170 |
| AF | -0.087±0.055 | -0.063±0.062 |  | -0.055±0.267 | +0.594±0.361 |  | -0.368±0.269 | +0.311±0.360 |
| DS-F | -0.004±0.021 | -0.022±0.028 |  | -0.091±0.107 | +0.157±0.160 |  | -0.086±0.114 | +0.053±0.165 |
| DS-B | +0.001±0.023 | +0.014±0.028 |  | +0.083±0.103 | +0.023±0.142 |  | +0.102±0.112 | +0.094±0.158 |
| CDT | -0.014±0.018 | +0.011±0.022 |  | +0.013±0.059 | -0.036±0.084 |  | -0.039±0.062 | +0.003±0.087 |
| Log_e_ (TRAILS A) | -0.002±0.004 | +0.009±0.007 |  | +0.026±0.016* | -0.009±0.029 |  | +0.019±0.017 | +0.032±0.029 |
| Log_e_(TRAILS B) | +0.002±0.007 | +0.001±0.010 |  | +0.030±0.030 | -0.027±0.047 |  | +0.033±0.032 | -0.020±0.053 |
|  |  |  |  |  |  |  |  |  |
| ***MODEL 2*** |  |  |  |  |  |  |  |  |
| **V1 NFL** |  |  |  |  |  |  |  |  |
| Normalized MMSE | -0.137±0.206 | -0.276±0.260 |  | +0.752±0.747 | +0.339±1.024 |  | +0.129±0.791 | -1.842±1.093* |
| CVLT-List A | -0.041±0.091 | -0.049±0.103 |  | +0.553±0.358^b^ | +0.406±0.555^b^ |  | +0.288±0.413 | -0.100±0.596 |
| CVLT-DFR | -0.049±0.041^b^ | +0.028±0.049^b^ |  | +0.299±0.177*^b^ | +0.103±0.230^b^ |  | +0.067±0.181 | +0.137±0.255 |
| BVRT | +0.048±0.064 | +0.037±0.082 |  | -0.367±0.253 | +0.329±0.400 |  | -0.096±0.266 | +0.734±0.398* |
| BTA | -0.005±0.032 | -0.094±0.044** |  | +0.032±0.125 | +0.215±0.203 |  | -0.037±0.123 | -0.282±0.200 |
| AF | -0.038±0.062 | +0.075±0.073 |  | -0.125±0.308 | -0.318±0.414 |  | -0.355±0.320 | -0.003±0.426 |
| DS-F | +0.004±0.024 | -0.044±0.033 |  | +0.133±0.120 | -0.110±0.163 |  | +0.156±0.132 | -0.377±0.175** |
| DS-B | +0.008±0.026 | -0.079±0.033** |  | -0.033±0.113 | +0.119±0.152 |  | -0.048±0.125 | -0.285±0.168* |
| CDT | +0.005±0.020 | -0.042±0.026 |  | +0.035±0.070 | +0.055±0.098 |  | +0.055±0.074 | -0.118±0.106 |
| Log_e_ (TRAILS A) | -0.002±0.005 | +0.001±0.008 |  | +0.026±0.018 | +0.009±0.033 |  | +0.020±0.020 | +0.014±0.033 |
| Log_e_(TRAILS B) | +0.000±0.008 | -0.007±0.012 |  | +0.005±0.034 | +0.039±0.053 |  | +0.013±0.036 | +0.003±0.060 |
|  |  |  |  |  |  |  |  |  |
| **δNFL** |  |  |  |  |  |  |  |  |
| Normalized MMSE | -0.059±0.194 | -0.131±0.220 |  | +0.780±0.630 | +0.912±0.887 |  | +0.947±0.677 | +0.415±0.918 |
| CVLT-List A | +0.069±0.082 | +0.019±0.085 |  | -0.625±0.300** | +0.651±0.459 |  | -0.349±0.348 | +0.767±0.498 |
| CVLT-DFR | +0.098±0.037*** | -0.010±0.040 |  | -0.380±0.147** | +0.189±0.190 |  | +0.029±0.153 | +0.159±0.215 |
| BVRT | +0.058±0.058 | -0.046±0.070 |  | +0.041±0.215 | -0.342±0.345 |  | +0.236±0.223 | -0.547±0.339 |
| BTA | -0.013±0.029 | -0.005±0.036 |  | +0.105±0.104 | +0.040±0.163 |  | +0.075±0.103 | +0.022±0.171 |
| AF | -0.095±0.057* | -0.072±0.063 |  | -0.070±0.261 | +0.458±0.358 |  | -0.391±0.267 | +0.087±0.361 |
| DS-F | -0.001±0.022 | -0.010±0.029 |  | -0.099±0.102 | +0.123±0.142 |  | -0.082±0.110 | +0.049±0.152 |
| DS-B | -0.005±0.024 | +0.016±0.028 |  | +0.107±0.095 | +0.011±0.131 |  | +0.101±0.104 | +0.069±0.143 |
| CDT | -0.012±0.018 | +0.019±0.022 |  | -0.003±0.059 | -0.053±0.085 |  | -0.039±0.063 | +0.031±0.090 |
| Log_e_ (TRAILS A) | -0.001±0.004 | +0.005±0.007 |  | +0.029±0.016* | -0.001±0.029 |  | +0.025±0.017 | +0.023±0.028 |
| Log_e_(TRAILS B) | +0.005±0.007 | +0.004±0.010 |  | +0.029±0.028 | -0.045±0.046 |  | +0.043±0.030 | -0.025±0.051 |

*Abbreviations:* AF=Animal Fluency; BTA=Brief Test of Attention; BVRT=Benton Visual Retention Test; CDT=Clock Drawing Test; CES-D=Center for Epidemiologic Studies-Depression; CVLT-DFR=California Verbal Learning Test-Delayed Free Recall; CVLT-List A=California Verbal Learning Test-List A; DS-B=Digits Span-Backward; DS-F=Digits Span-Forward; HEI-2010=Healthy Eating Index, 2010 version; MMSE=Mini-Mental State Examination; k=number of observations/participant; SD=Standard Deviation; SE=Standard Error; NfL=Neurofilament Light; TRAILS A=Trailmaking Test, Part A; TRAILS B=Trailmaking Test, Part B; WRAT-3 = Wide Range Achievement Test, 3rd revision; X = mean.

^a^ Models 1A.1-1K.2 included each of NfL (Log_e_ transformed, z-scored) or δNfL (annualized change in Log_e_ transformed NfL, z-scored), separately as the main predictor for v1 cognitive performance, cognitive change over time, and v2 cognitive performance (11 test scores), using a series of multiple linear mixed-effects and ordinary least square regression models, stratified by poverty status group. These models adjusted only for age, sex, race, poverty status, length of follow-up (years) (for models with follow-up outcome) and the inverse mills ratio. Models 2A.1-2K.2 followed a similar approach but adjusted further for selected socio-demographic, lifestyle and health-related factors, namely educational attainment, the WRAT-3 score, current drug use, current tobacco use, body mass index, self-rated health, co-morbidity index, HEI-2010, total energy intake, and the CES-D total score. 1 SD of baseline Log_e_(NfL) is estimated at 0.51; Mean=1.98. δNfL values are annualized changes in Log_e_ transformed NfL between v_1_ and v_2_, z-scored. 1 SD of annualized change in Log_e_(NfL) is estimated at 0.101 ; Mean=0.044.

^b^ p<0.05 for Poverty×NfL in models that are unstratified by race to which this 2-way interaction was included.

**p* < 0.10** *p* < 0.05; *** *p* < 0.010; *****p*<0.001, test for null hypothesis of γ=0 or β=0. Bolded values passed correction for multiple testing in Model 1.

**FIGURE S1. Summary of main findings by race, Model 1^a,b,c^**

*Abbreviations*: AA=African American; AF=Animal Fluency; BC=Baseline cognitive performance; BTA=Brief Test of Attention; BVRT=Benton Visual Retention Test; CC=Cognitive change; CDT=Clock Drawing Test; CVLT-DFR=California Verbal Learning Test-Delayed Free Recall; CVLT-List A=California Verbal Learning Test-List A; dNfL=z-scores of annualized rates of change NfL, Loge transformed; DS-B=Digits Span-Backward; DS-F=Digits Span-Forward; FC=Follow-up cognition; NfL_v1_=Plasma NfL levels, Log_e_ transformed, z-scored at v_1_; TRAILS A=Trailmaking Test, Part A; TRAILS B=Trailmaking Test, part B.

^a^ 1 SD of baseline Log_e_(NfL) is estimated at 0.51; Mean=1.98. 1 SD of annualized change in Log_e_(NfL) is estimated at 0.101 ; Mean=0.044. BVRT, TRAILS A and B are coded in the direction of higher score 🡪 poorer performance. All other test scores are in the direction of higher score 🡪 better performance.

^b^Cognitive tests were: 1. Normalized MMSE; 2.CVLT-List A; 3.CVLT-DFR;4.BVRT;5.BTA;6.AF;7.DS-F;8.DS-B;9.CDT;10.TRAILS A;11.TRAILS B.
